# Low Brain Levels of Dietary Polyphenols and Their Conjugates: Reassessing Mechanisms of Alzheimer’s Disease Prevention

**DOI:** 10.3390/jdad3020027

**Published:** 2026-06-01

**Authors:** Roshni Sharma, Kristina Shkirkova, William J. Mack, Rayudu Gopalakrishna

**Affiliations:** Department of Neurological Surgery, Keck School of Medicine, University of Southern California, Los Angeles, 90089, USA

**Keywords:** Alzheimer’s disease, amyloid-β, cAMP, epigallocatechin-3-gallate, glucuronide metabolites, laminin receptor, polyphenols, quercetin, quinone reductase 2, resveratrol

## Abstract

Dietary polyphenols such as quercetin, resveratrol, and (−)-epigallocatechin-3-gallate (EGCG) have shown neuroprotective effects in epidemiologic and experimental studies of Alzheimer’s disease (AD), although clinical evidence remains limited. This review highlights the importance of investigating glucuronide and sulfate conjugates of these polyphenols, as well as their intestinal microbial metabolites, at bioavailable low nanomolar concentrations, particularly those capable of reaching the brain. Although many in vitro studies use micromolar concentrations of aglycones, the relevance of such concentrations to neuroprotection remains uncertain. While polyphenols are redox-sensitive, their direct antioxidant or prooxidant effects may be limited at nanomolar concentrations. Instead, their neuroprotective actions appear to be mediated through high-affinity interactions with molecular targets such as the 67-kDa laminin receptor (67LR). This receptor binds both aglycones and conjugates at low nanomolar concentrations through a peptide G region containing glycosaminoglycan- and palindromic sequence-related motifs. The same region also binds the prion–amyloid-β complex, suggesting that polyphenols may antagonize amyloid-β binding and thereby prevent its neurotoxicity. The peptide G region may also function as a redox sensor. Binding of polyphenols to 67LR activates cAMP signaling and downstream neuroprotective pathways involving CREB, SIRT1, and protein phosphatase 2A. In addition, nanomolar concentrations of resveratrol and quercetin inhibit quinone reductase 2, an enzyme associated with cognitive decline and reported to be elevated in AD. Given their low bioavailability in the brain and their distinct molecular targets, combining multiple polyphenols at low doses may produce additive or synergistic effects, enhance efficacy, and minimize potential toxicity in the prevention of AD.

## Graphical Abstract

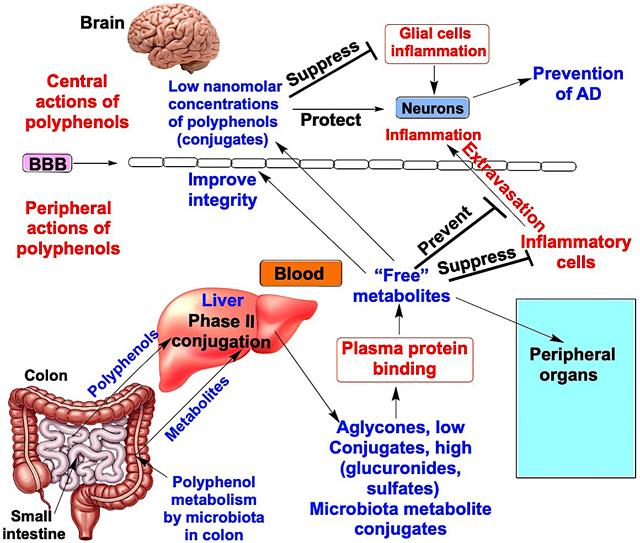

## 1. Introduction

Alzheimer’s disease (AD) is a major global health challenge and the most common age-related neurodegenerative disease. In 2021, an estimated 57 million people worldwide were living with dementia, with AD accounting for 60–70% of cases; nearly 10 million new dementia cases occur each year [1]. Based on current demographic trends, the global number of people living with dementia is projected to increase to 152.8 million by 2050 [2]. In the United States, an estimated 7.2 million adults aged 65 years and older are living with Alzheimer’s dementia in 2025, representing about 1 in 9 older adults (approximately 11%) [3].

Importantly, AD unfolds over a prolonged prodromal period, characterized by biological and cognitive alterations that precede clinical dementia by years [4]. During this trajectory, many individuals progress through amnestic mild cognitive impairment (aMCI), a transitional state carrying a 10–20% annual risk of conversion to AD [5]. This extended pre-dementia phase offers a critical therapeutic window for early interventions, notably lifestyle and dietary modifications [6]. Consequently, to develop effective preventative strategies, it is imperative to elucidate the mechanistic pathways through which dietary polyphenols exert their neuroprotective effects and inhibit AD pathogenesis.

### 1.1. Unique Focus of the Review

Given the clinical importance of AD, several reviews have addressed the relationship between polyphenols and AD from multiple perspectives [7–16]. This review focuses on the unique features of dietary polyphenols that limit their bioavailability, specifically, their rapid conjugation to glucuronide and sulfate metabolites, and the resulting low nanomolar concentrations reached in the brain. We emphasize the functional roles of these glucuronide and sulfate conjugates, as well as intestinal microbial metabolites. We also discuss the limitations of direct antioxidant and prooxidant mechanisms, highlighting instead the importance of high-affinity, redox-sensitive molecular targets for polyphenols. Although the human diet contains hundreds of polyphenols, this review focuses on quercetin, resveratrol, and (−)-epigallocatechin-3-gallate (EGCG), which have demonstrated consistent, reproducible neuroprotective effects in animal models of AD.

### 1.2. Multifactorial AD Pathogenesis

The β-amyloid (Aβ) hypothesis has long been a central framework for AD, proposing that abnormal amyloid precursor protein (APP) processing leads to the accumulation of pathogenic Aβ species [17–19]. Within this view, soluble Aβ oligomers are considered particularly toxic: they impair synaptic plasticity and memory-related signaling and can initiate downstream cascades that include tau dysregulation, neuroinflammation, and, ultimately, synaptic and neuronal loss [20,21]. Strong genetic support comes from autosomal-dominant (familial) AD, in which pathogenic variants in APP and presenilins alter Aβ generation and promote amyloid pathology [22]. Despite this biological rationale, translating Aβ-lowering into meaningful clinical benefit has proven difficult, with several approaches failing in symptomatic disease. More recently, however, anti-amyloid antibodies such as lecanemab and donanemab have produced a statistically significant, albeit modest, slowing of cognitive decline in early symptomatic AD. This supports Aβ as a valid target while also implying that timing (earlier intervention) and/or multi-target strategies may be necessary for larger clinical effects [23].

Currently, there is increasing emphasis on the multifactorial nature of AD. In addition to the important role of Aβ, other frameworks highlight contributions from tau pathology, chronic neuroinflammation, metabolic and mitochondrial dysfunction, dysregulated Ca^2+^ homeostasis, iron dyshomeostasis, and cerebrovascular or blood-brain barrier (BBB) impairment during disease initiation and progression [24–29]. This broader perspective has increased interest in combination and multi-target approaches that engage multiple disease mechanisms rather than focusing on a single pathway [24,30]. Within this context, dietary polyphenols have attracted attention because they can modulate several AD-relevant processes (e.g., Aβ- and tau-related pathways, inflammatory and oxidative signaling, and metabolic stress responses) across experimental systems [7,15].

### 1.3. Intervention of AD with Dietary Polyphenols

**Figure 1.**
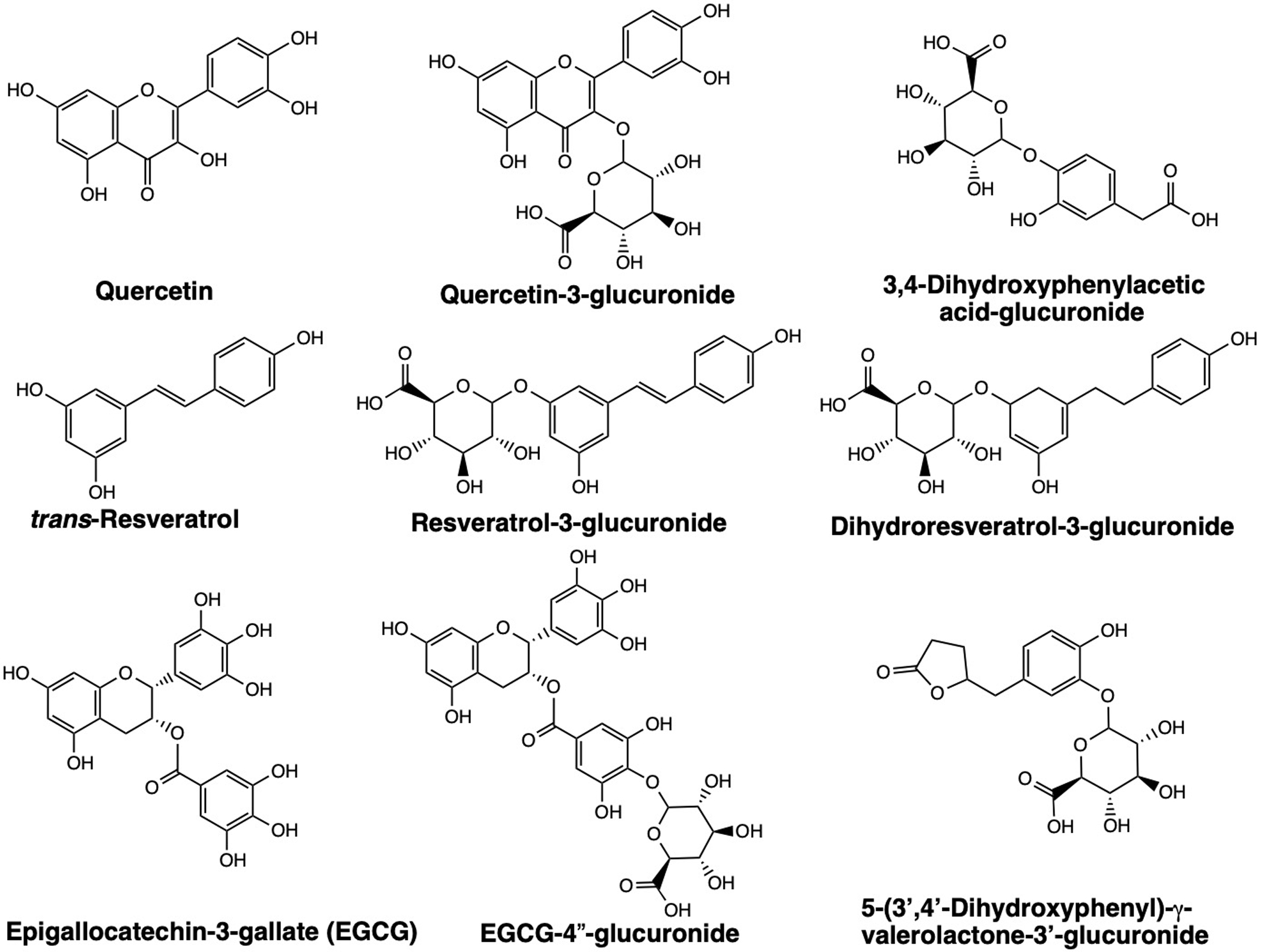
Structures of polyphenols aglycones (left three), glucuronide conjugates of polyphenols (middle three), and glucuronide conjugates of intestinal microbial metabolites (right three).

Epidemiological studies suggest that diets rich in plant-based foods are associated with slower cognitive decline [31], with several specific plant products shown to enhance memory [32,33]. Notably, the Mediterranean-DASH Intervention for Neurodegenerative Delay (MIND) diet is consistently linked to a lower risk of AD and related dementias [34–38]. This diet features foods high in polyphenols, such as fruits, vegetables, olive oil, and tea, alongside moderate amounts of red wine. Furthermore, several epidemiological studies have reported a lower incidence of dementia and AD specifically associated with green tea consumption [39,40]. In preclinical research, *trans*-resveratrol has been shown to attenuate AD pathology in various animal models [41–46], a protective effect similarly reported for quercetin in AD mouse models [47–52] (Figure 1). Resveratrol also protects isolated neurons from amyloid-β-induced toxicity and other harmful insults *in vitro* [53–56]. Finally, EGCG has demonstrated robust neuroprotective effects across multiple experimental models of AD [34,57,58].

### 1.4. Challenges in Understanding the Role of Dietary Polyphenols in AD Prevention

As extensively reviewed elsewhere, quercetin, resveratrol, and EGCG exert neuroprotective effects *in vitro* against Aβ toxicity and oxidative stress in neuronal cells; however, these effects often require 10 to 100 μM concentrations [59–62]. In contrast, these compounds reach only low nanomolar concentrations in the brain. These dietary polyphenols are absorbed to a limited extent and undergo rapid phase II metabolism to form glucuronide and sulfate conjugates [63–65]. Consequently, the parent (aglycone) compounds are present at very low concentrations in the plasma. Furthermore, both the parent compounds and their metabolites are extensively bound to plasma proteins [66–68]. As a result, only small amounts of aglycones and conjugates, often in the low nanomolar range, cross the BBB. This discrepancy is a critically important consideration when designing experiments to elucidate the mechanisms underlying their neuroprotective effects *in vivo*.

## 2. Absorption, Distribution, Metabolism, and Excretion of Polyphenols (ADME)

First, we reviewed the general ADME aspects of dietary polyphenols. We then examined these features for specific compounds, quercetin, resveratrol, and EGCG, summarizing the findings in Table 1. Finally, we explored polyphenol accumulation in the brain during prolonged supplementation, highlighting species-related differences in detected concentrations.

### 2.1. General Pharmacokinetic Aspects of Polyphenols

Dietary polyphenols exhibit limited and highly variable absorption; their bioavailability depends strongly on chemical structure, the food matrix, intestinal processing, and host factors [69–73]. In the small intestine, some low-molecular-weight aglycones and certain glycosides can be absorbed after hydrolysis. However, many larger, esterified, glycosylated, or polymerized polyphenols are poorly absorbed proximally. These compounds pass instead to the colon, where they undergo extensive microbial catabolism into smaller, absorbable phenolic metabolites [69–72]. During and after absorption, polyphenols undergo substantial first-pass metabolism in enterocytes and the liver. Glucuronidation, sulfation, and O-methylation produce the major circulating forms, meaning plasma typically contains conjugated metabolites rather than the parent aglycones [69–73]. Intestinal handling also involves efflux back into the lumen and interactions with carrier-mediated transport. Once in circulation, polyphenols are largely bound to plasma proteins, which influences their distribution, tissue uptake, and elimination [69,71,73]. Consequently, circulating concentrations of intact parent compounds remain low, transient, and far below those commonly used *in vitro*. Instead, conjugated and microbiota-derived metabolites constitute the principal forms that reach tissues and drive biological effects [70–73].

### 2.2. Quercetin

Quercetin is a flavonol-type flavonoid found predominantly in vegetables and fruits in the form of glycosides [92]. These glycosides are hydrolyzed by intestinal bacteria and by intestinal enzymes, such as lactase-phlorizin hydrolase and cytosolic β-glucosidase, to release the quercetin aglycone, which is then absorbed in the intestine [93]. Up to 20% of administered quercetin is absorbed, but its bioavailability is only 2% [74]. In the liver, quercetin is rapidly metabolized by phase II enzymes to form glucuronides, sulfates, mixed glucuronide-sulfate conjugates, and methylated glucuronides [64]. In plasma, only these conjugated metabolites are detected at nanomolar to low micromolar concentrations, depending on the dose of quercetin or quercetin glycosides administered to animals or humans; quercetin aglycone itself is not detectable [64]. In sheep administered dried onion skin, quercetin was detected in cerebrospinal fluid (CSF) at a concentration of 0.30 nM, whereas quercetin-3-O-glucuronide (Q-3-G) and other conjugated metabolites were not detectable [89] (Table 1). In contrast, Pasinetti and colleagues quantified Q-3-G in rat brain at concentrations as low as 1 nM following oral administration of a red wine polyphenol mixture [83].

Pharmacokinetic studies in humans receiving quercetin (500 mg, three times daily) indicate an average terminal plasma half-life of 3.5 h [94], while the elimination half-lives of its major conjugated metabolites range from 1.7 to 5.3 h [95]. Urinary excretion of intact quercetin metabolites accounts for only ~5% of the administered dose [95]. Instead, a significant portion of the compound undergoes extensive degradation, exhibiting an extended 'biological half-life' of 20 to 72 h, and is primarily recovered as expired CO_2_ [96].

### 2.3. Resveratrol

Resveratrol (3,5,4′-trihydroxystilbene) is a non-flavonoid polyphenol found in red wine, grapes, berries, and peanuts [97,98]. The *trans* isomer is the predominant and more stable form compared to its *cis* counterpart. In plant sources, resveratrol exists primarily in glycosylated forms. Upon ingestion, these glycosides are hydrolyzed by intestinal microbial glycosidases to release the absorbable aglycone [97,98]. Although nearly 75% of an oral dose is absorbed in the intestine, extensive first-pass metabolism reduces its absolute bioavailability to less than 1% [75]. Following the consumption of resveratrol-containing red wine or moderate doses of the purified compound (e.g., 25 mg) in humans, rapid metabolism renders the circulating aglycone largely undetectable; instead, glucuronide and sulfate conjugates are present at low concentrations [65,75,99]. Conversely, high pharmacological doses (100–5000 mg) yield detectable plasma levels of the free aglycone, albeit only in the nanomolar to low-micromolar range, with conjugate concentrations remaining significantly higher [75]. Because resveratrol crosses the BBB *in vitro* only to a limited extent [81], the parent compound and its metabolites achieve merely low nanomolar concentrations or remain undetectable in the brain and CSF [85].

Human studies indicate the plasma half-life of *trans*-resveratrol is approximately 1–3 h, whereas its conjugated metabolites exhibit a much longer half-life of about 9 h [100]. Consequently, while the parent compound is cleared rapidly, the pool of conjugates persists for an extended period [100]. Ultimately, most of the administered dose is recovered in the urine, predominantly as glucuronide and sulfate conjugates alongside microbiota-derived metabolites [75].

### 2.4. EGCG

EGCG is the most abundant catechin flavonoid in green tea, co-occurring with other catechins such as epicatechin, epigallocatechin, and epicatechin-3-gallate. Unlike polyphenols such as quercetin and resveratrol, which naturally occur primarily as glycosides, green tea catechins exist predominantly in the unconjugated (aglycone) form [101]. Following oral administration in humans and rodents, the systemic bioavailability of EGCG remains remarkably low, estimated at 0.1–0.3% [76]. However, its metabolic profile differs notably from other dietary polyphenols. When a green tea catechin mixture is administered to humans, a substantial fraction of EGCG (~45% or higher) circulates in the plasma as the intact aglycone, with the remainder present as conjugated metabolites, primarily EGCG-sulfate and EGCG-glucuronide [78]. Thus, compared to quercetin and resveratrol, EGCG undergoes relatively less extensive phase II conjugation, allowing a significant proportion to persist in its aglycone form in systemic circulation [78].

The extent to which EGCG and its metabolites penetrate the brain following green tea consumption or oral supplementation remains controversial. In one study, orally administered ^3^H-labeled EGCG produced measurable radioactivity in brain tissue, suggesting central nervous system (CNS) uptake; however, the specific chemical forms and absolute concentrations were not determined [[102]. Subsequent mouse models found that while EGCG distributed to several peripheral tissues after standard oral dosing, it remained undetectable in the brain [88]. In rats given 50 mg/kg of EGCG, brain concentrations of roughly 10 nM were reported [88]; yet, it is unclear whether residual blood contamination was adequately controlled for in this analysis. Conversely, another study achieved detectable brain EGCG levels of approximately 0.5 nmol/g in rats, but only following a very high pharmacological dose (500 mg/kg) [87].

*In vitro* models suggest limited BBB permeability for EGCG (Table 1). For context, human consumption of two cups of green tea yields a peak plasma EGCG concentration of approximately 0.2 μM [103]. Utilizing an *in vitro* system, Pervin et al. reported a BBB permeability rate of 5.6% for EGCG over a 1-h period, leading to an estimated delivery of nearly 10 nM to the brain parenchyma [82]. However, such *in vitro* models possess inherent limitations when predicting *in vivo* brain uptake. Highlighting this translational gap, clinical studies have found that neither EGCG nor its conjugates are detectable in human CSF following oral green tea consumption [91].

Pharmacokinetically, EGCG exhibits a plasma half-life of 3.4 h in humans following the ingestion of green tea or purified extracts [103]. Furthermore, only trace amounts of EGCG are excreted in the urine. Instead, elimination occurs predominantly via biliary secretion and fecal excretion, accompanied by extensive biotransformation into microbial metabolites within the gut [104].

### 2.5. Plasma Protein Binding and the “Free” Polyphenol Fraction

In plasma, these polyphenol aglycones are highly protein-bound: quercetin binding can reach 99% [66], resveratrol is nearly 98% bound (with ~50% binding for its conjugates) [67,79], and EGCG is 80–90% bound [68]. As demonstrated with numerous drugs and steroid hormones, extensive protein binding reduces the unbound (free) fraction, thereby limiting tissue availability and narrowing the range of accessible molecular targets. However, protein binding exists in a dynamic equilibrium; the bound pool serves as a reservoir that can replenish the free fraction as the unbound compound is distributed, metabolized, or excreted. Furthermore, binding to plasma proteins can slow glomerular filtration, thereby extending the compound's half-life, and may stabilize polyphenols against auto-oxidation. Despite these pharmacokinetic benefits, extensive protein binding significantly restricts free polyphenol levels in tissues and further limits their penetration across the BBB. Consequently, brain exposure may remain low, compounding the separate permeation constraints already imposed by molecular size and polarity. Methodologically, it is also important to note that polyphenols and their metabolites are typically extracted from plasma using organic solvents, a process that yields total (bound plus unbound) concentrations rather than isolating the biologically active free fraction. Therefore, protein binding must be critically considered when interpreting *in vivo* exposure measurements and when designing translationally relevant *in vitro* experiments to elucidate mechanisms of action.

### 2.6. Polyphenol accumulation in the brain with chronic administration

While prolonged supplementation can enhance the brain accumulation of quercetin, resveratrol, and EGCG, the resulting concentrations remain low, and the strength of the evidence varies considerably among the three compounds [62,76,105–108]. In the case of quercetin, repeated oral dosing in rats yields a measurable accumulation of conjugated metabolites in the brain, though the parent aglycone remains undetectable [105]. Specifically, feeding rats a purified diet containing 1% (w/w) quercetin aglycone (equivalent to a 213 mg intake) produced tissue concentrations of nearly 20 pmol/g after one hour, doubling to approximately 40 pmol/g after one month of feeding [105]. Similarly, animal models indicate that repeated administration of EGCG increases its cerebral accumulation; however, absolute levels remain low and may partially represent EGCG-derived metabolites rather than intact parent molecules [62,76]. Conversely, while prolonged resveratrol supplementation elicits sustained neurobiological effects, direct evidence for the progressive accumulation of the parent compound in brain tissue is less robust. This is largely due to its rapid metabolism and the inherently low brain availability associated with conventional systemic dosing [107,108].

### 2.7. Species-dependent variation in the accumulation of polyphenols in the brain

Species-dependent differences significantly influence the brain accumulation of quercetin, resveratrol, and EGCG. In the case of quercetin, while rats, mice, and humans all circulate predominantly conjugated metabolites rather than the free aglycone, the specific proportions of glucuronidated, sulfated, and methylated forms vary across species, potentially impacting brain penetrance and retention [71,109]. Similarly, although resveratrol undergoes extensive first-pass metabolism across all three species, marked differences in glucuronidation, sulfation, clearance rates, and half-lives suggest that rodents are not strictly interchangeable models for predicting human CNS exposure [71,109]. Regarding EGCG, mice appear to achieve higher systemic exposure than rats and may more closely mirror human bioavailability, indicating that rat models could underestimate actual exposure levels [76,110]. Because direct data on human brain tissue are inherently limited, these interspecies comparisons rely heavily on plasma pharmacokinetics, circulating metabolite profiles, and the concentrations of polyphenols and their specific metabolites in the CSF [71,111].

## 3. Polyphenol Aglycone Actions at Micromolar Concentrations

Collectively, pharmacokinetic studies suggest that polyphenols, such as quercetin, resveratrol, and EGCG, or their conjugates may reach the brain at low nanomolar concentrations, often falling below detection limits. Nonetheless, many *in vitro* studies demonstrating the beneficial effects of these polyphenols (including antioxidant and prooxidant activities, as well as the inhibition of β-amyloid aggregation) rely on much higher micromolar concentrations. While these observations provide valuable mechanistic insights, further research is required to determine whether such effects are achievable in the brain at physiologically bioavailable concentrations and in relevant chemical forms.

### 3.1. Antioxidants – Limitations

Although dietary polyphenols can, in principle, quench reactive oxygen species (ROS) through direct chemical scavenging, their contribution to antioxidant defense *in vivo*, particularly in the brain, is thought to be limited because their physiological concentrations typically remain in the low-nanomolar range [112–115]. In addition, polyphenol glucuronide and sulfate conjugates generally exhibit weaker radical-scavenging activity than their corresponding aglycones [92,116]. Instead, polyphenols such as quercetin, resveratrol, and EGCG, whether as aglycones or as glucuronide/sulfate metabolites, can modulate redox-sensitive signaling pathways that upregulate the expression of endogenous antioxidant enzymes [117–119]. Compounds acting primarily through this mechanism are termed “indirect antioxidants”: rather than directly scavenging ROS, they enhance intrinsic antioxidant defenses, conferring protection that may persist even after the compound has been cleared from the tissues [120,121].

### 3.2. Prooxidants – Limitations

Conversely, several studies have described how quercetin, resveratrol, and EGCG can act as prooxidants under conditions that favor their oxidation or transition-metal redox cycling, leading to net ROS generation rather than ROS quenching. For example, copper complexes of quercetin and resveratrol can promote ROS formation and oxidative DNA damage [122,123]. However, at lower, non-toxic exposures, this prooxidant activity may function as a mild oxidative stimulus that engages adaptive defenses (e.g., Nrf2-dependent gene expression), consistent with a hormetic mechanism [124]. Notably, H_2_O_2_ generated via EGCG autoxidation has been reported to be protective in human keratinocytes [125]. The prooxidant actions of EGCG have thus been proposed as a potential contributor to its health benefits, though they may also mediate adverse effects under certain conditions [126]. Furthermore, Forman and colleagues have detailed how electrophiles derived from dietary polyphenols can increase nucleophilic tone by activating Nrf2 [127], and Shah et al. demonstrated the critical role of Nrf2 and heme oxygenase-1 in flavanol-mediated neuroprotection [128].

Halliwell and colleagues have cautioned that cell culture media can generate artifacts, as iron-dependent oxidation can produce reactive species such as H_2_O_2_ and quinones or semiquinones [129]. They also noted that the potential prooxidant activities of polyphenols in humans, particularly at the low concentrations achieved through diet, remain to be clearly established *in vivo* [130]. While quinone metabolites of these polyphenols have not been isolated from animals consuming typical dietary amounts, they may form transiently and react rapidly with low-molecular-weight thiols. Furthermore, although EGCG, quercetin, and resveratrol form adducts with protein thiols *in vitro*, these specific adducts have not been observed *in vivo* at typical dietary intakes [131,132]. In contrast, EGCG-quinone-cysteine thiol adducts have been isolated from the urine of mice administered high, toxic doses of EGCG, but not following lower, dietary-relevant doses [133]. However, minor amounts of a quercetin-glutathione adduct have been identified in human plasma following the consumption of quercetin-rich foods [134]. Rapid conjugation by phase II enzymes likely limits quinone formation, thereby minimizing the potential for toxicity often observed *in vitro*. Nevertheless, the trace amounts of quinones that do form may react with signal transduction enzymes, oxidize vicinal thiols, and subsequently revert to their phenolic states. Consequently, these transiently formed quinones could play a functional role in cellular signaling.

### 3.3. Inhibition of Protein Kinases and Other Enzymes – Limitations

Previous reviews have detailed resveratrol’s capacity to inhibit multiple enzymes—including cyclooxygenase, lipoxygenase, PKCs, ERK1, JNK1, p38, Src, ribonucleotide reductase, DNA polymerases, PKD, and aromatase—typically exhibiting IC_50_ values in the 10–60 μM range [135]. Similarly, EGCG has been shown to inhibit various receptor tyrosine kinases at effective concentrations generally ranging from 5 to 100 μM [136], as well as cyclooxygenase-1 and −2 with IC_50_ values of 17–28 μM [137]. However, given that polyphenol aglycones are present at substantially lower concentrations in plasma and tissues, the physiological relevance of these high-dose *in vitro* findings remains uncertain.

### 3.4. Inhibition of Aβ Aggregation – Limitations

*In vitro*, several dietary polyphenols can directly interfere with Aβ self-assembly. For instance, quercetin inhibits Aβ fibril formation and can promote the disaggregation of preformed fibrils, at least in peptide/fibril model systems [138]. Resveratrol inhibits Aβ42 fibril formation and reduces Aβ-associated cytotoxicity, although it may not completely prevent oligomer formation [139]. Furthermore, EGCG can bind natively unfolded Aβ, redirecting its aggregation away from β-sheet-rich fibrils and toward unstructured, off-pathway oligomers [140]. However, these inhibitory effects are typically observed at micromolar concentrations of the parent aglycones. It remains to be determined whether comparable Aβ-polyphenol interactions occur within the brain at the low nanomolar concentrations achievable *in vivo*, either for the aglycones or their conjugated metabolites.

## 4. Polyphenol Metabolites Formed by Gut Microbiota, Gut-brain Axis

Given that parent polyphenol aglycones circulate at very low levels, attention has shifted to the gut microbiota's role in their metabolism to help explain their health benefits. Because many polyphenols are poorly absorbed in the small intestine, a substantial fraction reaches the colon, where the microbiota converts them into smaller, more readily absorbable molecules [141]. Specifically, quercetin is degraded via C-ring fission and side-chain modifications into more absorbable phenolic acids, such as 3,4-dihydroxyphenylacetic acid [142,143]. Similarly, gut microbes reduce resveratrol to dihydroresveratrol and, depending on the individual’s microbiota, can further convert it to dehydroxylated products like lunularin [144,145]. For EGCG, microbial esterases first catalyze its degalloylation, yielding epigallocatechin and gallic acid [146–148]. Epigallocatechin is subsequently metabolized into downstream products, including valerolactone-type metabolites [146–148]., while gallic acid can be converted into smaller phenolics like pyrogallol, which notably induces Nrf2-associated gene expression more potently than its parent compound, EGCG [149]. Ultimately, metabolite profiles and yields vary substantially among individuals, reflecting interindividual differences in microbiota composition and metabolic capacity.

Growing evidence suggests that these microbial metabolites contribute significantly to the health benefits associated with dietary polyphenols. However, many of these gut-derived products are themselves phenolic compounds that undergo extensive phase II metabolism in the liver to form glucuronide and sulfate conjugates. This metabolic reality reiterates the fundamental question of how these specific conjugates and intestinal microbial metabolites exert their biological effects. Consequently, defining the mechanisms by which microbiota-derived metabolites and their conjugates support neuroprotection via the gut-brain axis in AD remains a critical research priority.

## 5. Glucuronide and Sulfate Conjugates as Active Metabolites

Given the low circulating concentrations of parent polyphenols compared to the relatively higher levels of their conjugates, researchers increasingly propose that glucuronide and sulfate metabolites may mediate at least a portion of the biological actions attributed to the parent compounds [150]. Traditionally, drug conjugates were viewed merely as inactive excretory products; however, this paradigm is shifting as a growing number of compounds are shown to retain or gain biological activity in their conjugated forms. For instance, morphine is activated via glucuronidation, while the antihypertensive drug minoxidil is activated by sulfation [151,152]. Notably, morphine-6-glucuronide is actively transported across the BBB and can also be synthesized locally within the brain, where it directly contributes to analgesia [153,154].

While direct evidence indicates that glucuronide conjugates of both resveratrol and quercetin can reach CNS compartments, their net brain exposure remains generally low. Because these glucuronides are polar anions, they do not readily cross the BBB via passive diffusion. Consequently, when these metabolites are detected in the CSF or brain tissue, their presence is presumed to reflect carrier-mediated transport processes at brain barrier sites [155]. Furthermore, a fraction of circulating polyphenol glucuronides may be taken up by BBB-associated cells and locally deconjugated into aglycones. Since brain tissue also expresses UDP-glucuronosyltransferases and sulfotransferases, these aglycones can subsequently undergo local reconjugation within the neural environment [156–159].

An alternative hypothesis posits that polyphenol glucuronides and sulfates function primarily as circulating reservoirs. In this model, these conjugates are locally hydrolyzed by β-glucuronidase and sulfatase enzymes, regenerating the corresponding aglycones within target tissues to exert their biological effects [160,161]. This targeted deconjugation may occur more readily at sites of inflammation, which are characterized by an increased cellular release of these hydrolases [162,163]. Nevertheless, this process would still yield only minute quantities of free aglycones, raising the critical question of how such low local concentrations can mediate significant biological outcomes.

## 6. Direct Protective Actions of Polyphenols on Neurons – Binding to High-affinity Receptors and Relevance to AD Prevention

Although oral administration yields only low nanomolar concentrations of polyphenols in the brain, these compounds may still exert neuroprotective effects by acting directly on neurons via high-affinity receptor binding. Two relevant molecular targets are the 67-kDa laminin receptor (67LR) and quinone reductase 2 (QR2). Both respond to polyphenols at these low concentrations and may contribute to the prevention of AD.

### 6. 1. Neuroprotective Actions of Polyphenols Mediated by 67LR

#### 6.1.1. 67LR as an Evolutionarily Conserved Multifunctional Protein: Friend or Foe

The 67LR is a cell-surface laminin receptor originally implicated in tumor cell adhesion, invasion, and metastasis [164–169]. Evolutionarily conserved, this receptor is initially synthesized as a 37-kDa precursor (37LRP), also known as ribosomal protein SA (RPSA), which plays an essential role in ribosomal biogenesis and protein synthesis [168,170]. Through mechanisms that remain incompletely understood, this 37-kDa precursor converts into the mature 67-kDa cell-surface form, functioning as a high-affinity laminin receptor [167–169]. Furthermore, 67LR mediates the internalization of diverse pathogenic bacteria and viruses, as well as cellular and infectious prion proteins [167–169]. Crucially, it also serves as a coreceptor for the Aβ-prion protein complex, facilitating Aβ internalization and inducing neuronal death, a critical step in the pathogenesis of AD [171,172]. Collectively, these findings indicate that 67LR plays multiple pathological roles.

The widespread tissue distribution of 67LR, particularly within the brain [173], suggests its involvement in essential cellular processes far beyond cancer metastasis and infections. Indeed, we and others have demonstrated that 67LR mediates several beneficial physiological functions, including neuritogenesis, neuroprotection, and the maintenance of BBB integrity [174–177]. Recent studies further highlight its importance in the axonal growth of dorsal root ganglion neurons [178], while other research indicates it mediates pigment epithelium-derived factor-induced morphogenesis in cortical neurons [179]. Furthermore, gene knockout studies show that the homozygous deletion of *Rpsa* (which encodes 37LRP) results in embryonic lethality, underscoring its indispensable role in early development [167]. Ultimately, 67LR can exert either pathological or protective effects depending strictly on the specific ligands it binds or internalizes.

#### 6.1.2. 67LR as a High-affinity Target for Dietary Polyphenols and Their Metabolites

Tachibana and colleagues identified the high-affinity binding of EGCG (*K*d = 40 nM) to 67LR, demonstrating that this receptor mediates cancer cell death at low micromolar concentrations of EGCG [180,181]. This discovery stemmed from an unbiased screening strategy designed to identify proteins upregulated by retinoic acid that sensitize cancer cells to EGCG-induced apoptosis [180]. Strikingly, in contrast to the apoptotic effects observed at micromolar levels, EGCG promotes neuroprotection and neuritogenesis at low nanomolar concentrations, also in a strictly 67LR-dependent manner [174,175,182,183].

**Figure 2.**
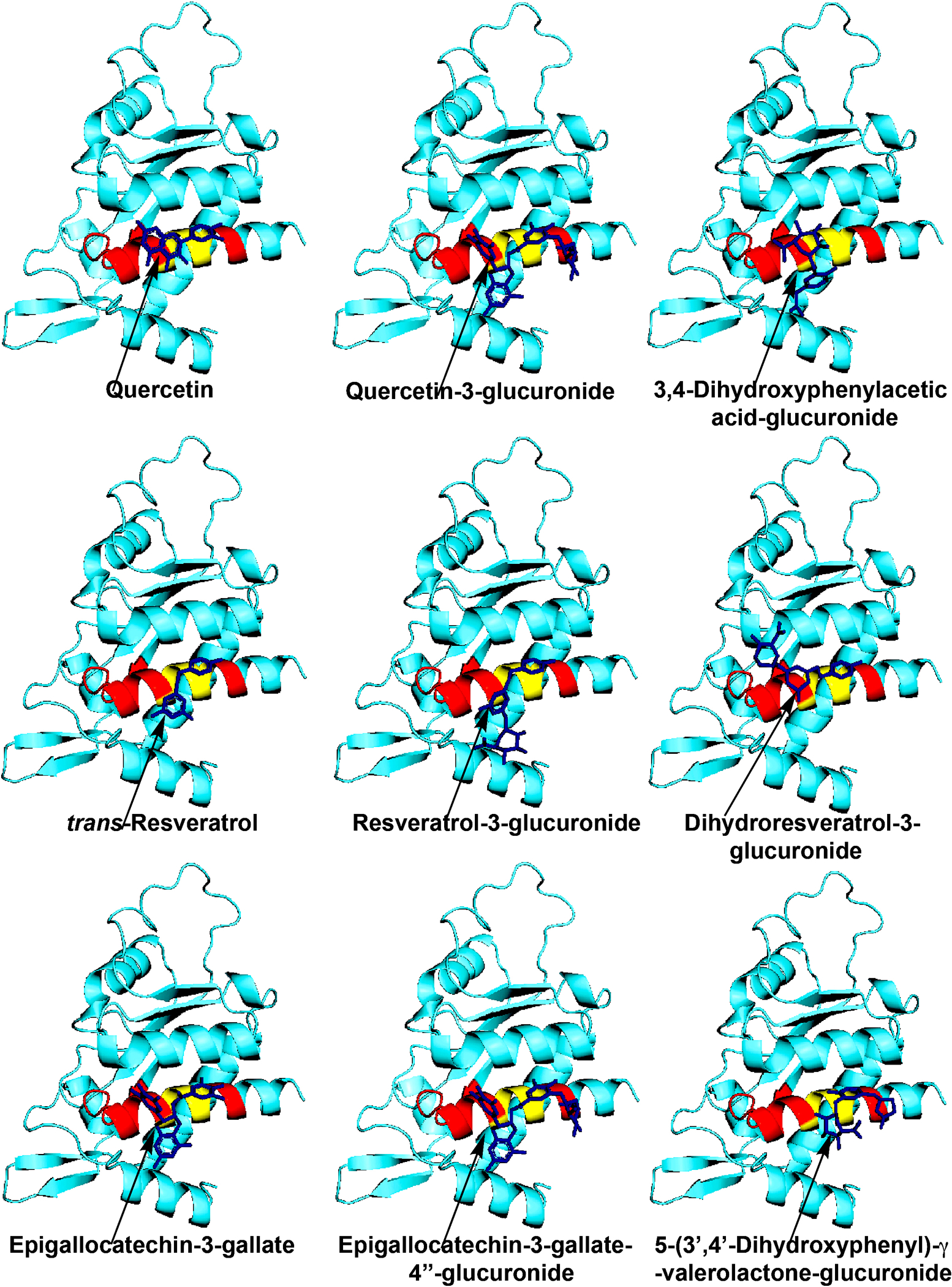
Molecular docking of polyphenols and their metabolites to truncated 37LRP. PyMOL-generated models showing the crystal structure of truncated 37LRP (cyan) docked with polyphenols and their glucuronide metabolites. The peptide G region is highlighted in red, the palindromic sequence in yellow, and the bound ligands in blue. The docked ligands are grouped as follows: polyphenol aglycones (left three), glucuronide conjugates of polyphenols (middle three), and glucuronide conjugates of intestinal microbial metabolites (right three).

Research from our laboratory and others demonstrates that EGCG binds to two distinct sites on 67LR, notably within the peptide G region [183,184]. Molecular docking studies indicate that the “A” site binds EGCG and other polyphenols with higher affinity. This site is primarily composed of the second half of the peptide G region, which features a hydrophobic palindromic sequence encompassing Trp175 and Trp176 (Figure 2, Table 2). Indeed, assays using synthetic peptide G show that EGCG binds this palindrome with high affinity (1.95 nM) [183]. In contrast, the “B” site involves residues from the first half of the peptide G region (outside the palindrome) and exhibits lower polyphenol binding affinity. Notably, other dietary polyphenols, including resveratrol and quercetin, also interact with 67LR via this region [182,183]. Furthermore, α-tocopherol and α-tocotrienol interact with 67LR through Trp176, reinforcing the role of the peptide G sequence as a shared binding motif [185,186]. A variety of other polyphenols, such as thearubigin, theaflavin, rutin, epicatechin-3-gallate, epigallocatechin, and procyanidin C1, also bind 67LR with high affinity to elicit cellular responses [187,188]. Together, these findings suggest that 67LR serves as a common receptor for a diverse array of dietary polyphenols.

Interestingly, the glucuronide and sulfate conjugates of these polyphenols exhibit higher binding affinity for peptide G than their aglycone forms [182–184]. Furthermore, molecular docking studies indicate that intestinal microbial metabolites of quercetin (3,4-dihydroxyphenylacetic acid), *trans*-resveratrol (dihydroresveratrol), and EGCG [5-(3′,4′-dihydroxyphenyl)-γ-valerolactone] also bind to 67LR, albeit generally with lower affinity than their corresponding polyphenol aglycones (Table 2). However, upon conjugation, the glucuronide forms of these microbial metabolites dock with higher affinity than their unconjugated counterparts. Notably, binding assays using isolated peptide G revealed a specific exception: dihydroresveratrol binds with higher affinity than its parent compound, *trans*-resveratrol [183]. Much like *trans*-resveratrol, dihydroresveratrol also induces elevations in intracellular cAMP via a strictly 67LR-dependent mechanism [183].

Many dietary polyphenols accumulate inside cells only to a limited extent because they are relatively polar and are subject to active efflux by membrane transporters [189]. Glucuronide and sulfate conjugates are even more hydrophilic organic anions; consequently, their cellular entry is often transporter-dependent, while efflux transporters can further restrict their intracellular exposure [190]. In this context, a cell-surface binding protein like 67LR is ideally positioned to sense extracellular polyphenols without requiring substantial intracellular accumulation. Collectively, these findings support the concept that 67LR serves as a common receptor for structurally diverse dietary polyphenol aglycones, their glucuronide conjugates, and intestinal microbial metabolites, thereby potentially mediating their broad biological and chemopreventive actions.

#### 6.1.3. 67LR Structural Basis for Binding of Polyphenol Glucuronide/Sulfate Conjugates

**Figure 3.**
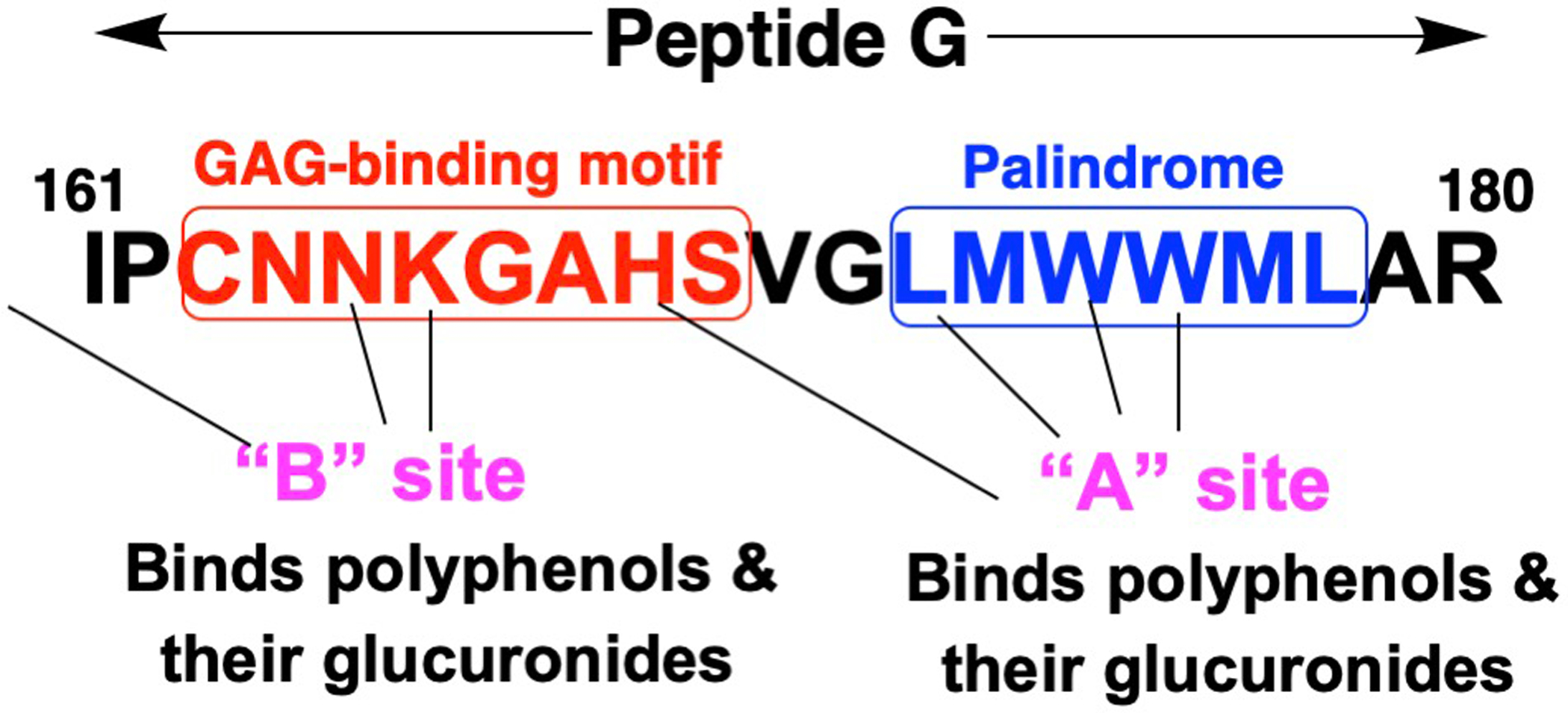
Binding sites “A” and “B” for polyphenols and their glucuronides within the peptide G region of 67LR. The peptide G region contains a palindromic sequence (blue) and a GAG-binding motif (red). The high-affinity “A” site mediates the binding of polyphenols and their glucuronides, primarily utilizing Leu173, Trp175, and Trp176 within the palindrome, alongside His169 from the GAG-binding motif and other residues. Conversely, the “B” site comprises Asn165 and Lys166 within the GAG-binding motif, along with additional amino acids, to facilitate ligand binding.

Glucuronide and sulfate conjugates of polyphenols are highly water-soluble and are thus generally expected to bind weakly to receptors. However, contrary to this long-held view, these polyphenol conjugates bind to 67LR with even higher affinity than their aglycones [182–184]. While the mechanisms by which other conjugates, such as morphine-6-glucuronide and minoxidil sulfate, bind their receptors with high affinity and exert enhanced biological activity remain unclear [151,152], 67LR possesses unique structural features that explain this phenomenon. Specifically, 67LR interacts with heparan sulfate proteoglycans, a class of glycosaminoglycans (GAGs) rich in uronic acids (D-glucuronic and L-iduronic acid) and sulfate residues [191]. GAG-binding motifs, typically following the consensus sequences X-B-B-X-B-X or X-B-B-B-X-X-B-X (where B is a basic amino acid and X is a hydropathic residue), are well-documented in other proteins [192,193]. The peptide G region of 67LR contains one such GAG-binding motif, which likely provides the additional interaction coordinates necessary to facilitate the high-affinity binding of polyphenol conjugates at both the A and B sites (Figure 3).

While our primary focus is the binding of polyphenols to mature, cell-surface 67LR, crystallographic data for this specific form remain unavailable. Instead, existing structural data are restricted to the bacterial recombinant precursor, 37LRP. We acknowledge that the conformation of mature 67LR may differ from that of 37LRP, which could potentially alter its binding affinity for polyphenols. Furthermore, while other groups have utilized surface plasmon resonance to measure the affinity of EGCG for the receptor [180], our study employed a synthetic peptide G in binding assays [182,183]. Nevertheless, our conclusion that polyphenols bind cell-surface 67LR with high affinity is strongly supported by the observation that 67LR-blocking antibodies inhibit the cellular effects of these compounds and their metabolites at low nanomolar concentrations. Ultimately, future studies directly investigating polyphenol binding to the intact 67LR protein are warranted.

#### 6.1.4. 67LR as a Coreceptor for the Aβ-prion Complex – Neuronal Cell Death

Paradoxically, while 67LR serves as a common receptor for a diverse array of neuroprotective dietary polyphenols, it also functions as a co-receptor for neurotoxic amyloid-beta (Aβ) oligomers. Aβ neurotoxicity is largely driven by the high-affinity binding of these Aβ oligomers (AβOs) to neuronal cell-surface receptors [194,195]. Strittmatter and colleagues identified that the lipid raft-associated cellular prion protein (PrP^C^) is a major high-affinity receptor for AβOs [196,197]. This AβO-PrP^C^ complex can subsequently interact with various co-receptors to mediate neuronal toxicity [194,195]. Building on this, Weiss and colleagues identified 67LR, which also localizes to lipid rafts, as a specific co-receptor for the AβO-PrP^C^ complex [171,172]. Although PrP^C^ is known to bind 67LR directly via the peptide G region [191], the direct binding of the intact AβO-PrPC complex to 67LR remains unconfirmed. Functionally, 67LR facilitates the internalization of this neurotoxic complex, driving intracellular Aβ accumulation and subsequent neuronal cell death [171,172]. Consequently, 67LR presents a promising therapeutic target, as evidenced by reduced neurodegeneration in AD transgenic mice following the intranasal administration of a 67LR-blocking antibody [198].

The binding of Aβ oligomers to cell-surface receptors triggers their internalization, leading to increased ROS generation, synaptic toxicity, and neuronal death [194,195,199]. Therefore, elucidating why polyphenol-induced signaling diverges from this pathway, and how it effectively counteracts these detrimental effects, represents a critical area of ongoing research.

#### 6.1.5. 67LR-mediated cAMP Signaling and Downstream Events for AD Prevention by Dietary Polyphenols

We previously reported a rapid, several-fold increase in intracellular cAMP levels within neuronal cells treated with low nanomolar concentrations of resveratrol, EGCG, and quercetin [182,183]. This elevation was prevented by a 67LR-blocking antibody, indicating that 67LR mediates cAMP induction by these polyphenols [183]. Because 67LR is not a G-protein-coupled receptor, it cannot directly activate adenylyl cyclase (AC) [168]. Although resveratrol has been reported to inhibit phosphodiesterases (PDEs), a mechanism that could elevate intracellular cAMP levels, this effect requires relatively high concentrations (20–40 μM) [200]. Consistent with this, we did not observe the inhibition of PDE4B or PDE4D at low nanomolar concentrations of these polyphenols.

Compared with healthy age-matched controls, patients with AD and AD mouse models exhibit markedly reduced levels of cAMP, AC, and PKA in the hippocampus [201]. The cAMP-elevating neuroprotective peptide, pituitary adenylyl cyclase-activating polypeptide (PACAP), protects neurons from Aβ oligomer (AβO)-induced cell death [202]; however, PACAP levels are reduced in AD patients relative to age-matched controls [203]. Furthermore, the administration of PACAP improves cognitive performance in AD transgenic mice [204]. Phosphodiesterase (PDE) inhibitors are currently under clinical investigation for AD treatment [205]; yet, chronic, non-physiological activation of cAMP signaling by these agents can lead to cognitive impairment, hyperexcitability, and hyperalgesia [206]. In contrast, dietary polyphenols induce modest, localized increases in intracellular cAMP, potentially avoiding the adverse effects associated with synthetic PDE inhibitors. However, the precise mechanisms by which 67LR binding enhances cAMP generation remain unclear.

**Figure 4.**
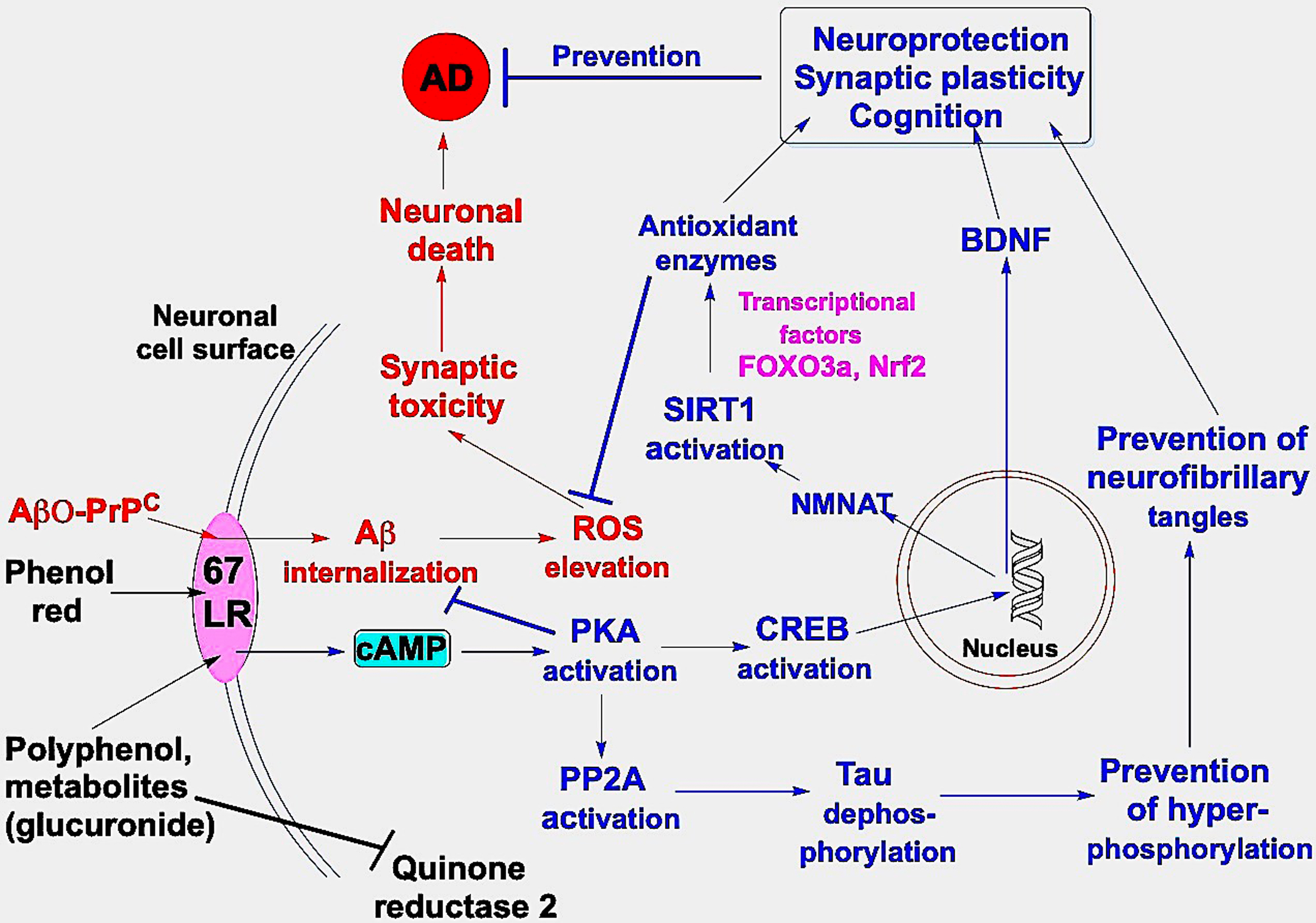
The Aβ-PrP^c^ complex binds to 67LR to trigger signaling that promotes neuronal death, whereas polyphenols and their glucuronide metabolites compete for the same receptor to activate neuroprotective pathways. Binding of the Aβ-PrP^c^ complex to 67LR promotes Aβ internalization, elevates reactive oxygen species (ROS), induces synaptic toxicity, and ultimately drives neuronal death and Alzheimer's disease (AD) progression (events shown in red). In contrast, polyphenols and their conjugates bind to 67LR and increase intracellular cAMP, thereby activating PKA and subsequently phosphorylating CREB. Activated CREB promotes the transcription of BDNF and NMNAT (nicotinamide mononucleotide adenylyl transferase). NMNAT then supports SIRT1 activation, which deacetylates transcription factors such as FOXO3a and Nrf2, inducing antioxidant enzymes that reduce ROS levels. Additionally, PKA activates protein phosphatase 2A (PP2A), which dephosphorylates tau to limit its hyperphosphorylation and the formation of neurofibrillary tangles. Together, these events (shown in blue) promote neuroprotection and neuroplasticity, improve cognition, and may slow AD progression. Furthermore, polyphenols such as resveratrol and quercetin inhibit quinone reductase 2 (QR2), an enzyme elevated in AD and linked to cognitive decline.

As illustrated in Figure 4, cAMP levels rise in response to dietary polyphenols and activate protein kinase A (PKA). PKA then phosphorylates the transcription factor cAMP- response element-binding protein (CREB) at Ser133, which increases the expression of brain-derived neurotrophic factor (BDNF), a neurotrophin essential for neuronal survival [207–209]. Consistent with this, enhanced CREB activation has been reported in neurons treated with low concentrations of quercetin-3-glucuronide [83]. Furthermore, cAMP signaling enhances the activity of sirtuin 1 (SIRT1), an NAD^+^-dependent deacetylase [210]. The cAMP/CREB pathway upregulates nicotinamide phosphoribosyltransferase (NAMPT), thereby raising the intracellular NAD^+^ levels required for optimal SIRT1 activity [211]. Once activated, SIRT1 deacetylates and stimulates key transcriptional regulators, including PGC-1α (involved in mitochondrial biogenesis and antioxidant defense) and FOXO3 (which regulates oxidative stress responses and longevity) [212,213]. SIRT1 also deacetylates and inhibits NF-κB to reduce inflammation, an effect that may benefit cognitive function [214]. Resveratrol is widely documented to increase SIRT1 expression and activity, protecting against Aβ toxicity in experimental AD models [215,216].; quercetin and EGCG reportedly share this SIRT1-activating capacity [217,218]. However, it remains to be determined whether the low concentrations of polyphenol aglycones and conjugates actually achieved in the brain are sufficient to activate neuronal SIRT1 *in vivo*. Ultimately, both BDNF and SIRT1 protect neurons from Aβ-induced toxicity while enhancing synaptic plasticity and cognitive function [54,219]. Because aging naturally dysregulates cAMP-PKA signaling [220], polyphenol-induced enhancement of this pathway offers a highly beneficial countermeasure.

Once activated by polyphenol treatment, the cAMP/PKA pathway phosphorylates and activates protein phosphatase 2A (PP2A), a key serine/threonine phosphatase [221,222]. This step is critical, as PP2A is the primary enzyme responsible for dephosphorylating the neuronal microtubule-associated protein tau [223]. By preventing tau hyperphosphorylation, PP2A activation inhibits the formation of neurofibrillary tangles and subsequent neuronal death. This mechanism is especially relevant to AD, given that PP2A activity is markedly reduced in the brains of AD patients [224]. Consequently, the activation of cAMP signaling by polyphenols via the 67LR receptor may represent a central mechanism driving their neuroprotective effects and their capacity to mitigate AD pathology.

#### 6.1.6. Potential Antagonism Between AβO-PrP^C^ and Dietary Polyphenols at 67LR

Because the AβO-PrP^c^ complex, as well as polyphenols and their metabolites, bind to the same peptide G region of 67LR [182,183,191], they likely compete for this site. Consequently, polyphenols may antagonize AβO-PrP^c^ binding, thereby preventing Aβ internalization and reducing neurotoxicity. Furthermore, polyphenol binding to 67LR induces cAMP/PKA signaling, which activates CREB, SIRT1, and PP2A. As illustrated in Figure 4, this cascade elevates the activity of antioxidant enzymes to promote neuroprotection and synaptic plasticity. Additionally, elevated cAMP has been shown to independently reduce Aβ internalization [199]. Consistent with this mechanism, other studies have demonstrated that cAMP protects cortical neurons from AβO-induced cell death [225].

#### 6.1.7. Phenol Red Binds to 67LR and Interferes with Polyphenol and Aβ-PrP^C^ Interactions

Phenol red, a widely used pH indicator in cell culture media, binds with high affinity to the peptide G region of 67LR. Because polyphenols and the Aβ-PrP^c^ complex target this exact site, phenol red can significantly interfere with their binding. Standard culture media, such as DMEM, RPMI 1640, and Neurobasal, contain phenol red at concentrations as high as 42, 14, and 21 μM, respectively. Consequently, these high background concentrations necessitate artificially high polyphenol doses to achieve neuroprotective effects *in vitro*. Initially unaware of this interference, we found that high nanomolar concentrations of EGCG were required to elicit neuritogenic and neuroprotective effects in phenol red-containing media [174,175]. However, after culturing cells in phenol red-free medium for two to three passages, we demonstrated that as little as 5 nM EGCG was sufficient to produce neuroprotection [183]. Similarly, we previously observed that omitting phenol red decreased the concentration of Aβ required to induce neuronal cell death [199]. Although direct competition between phenol red and Aβ oligomers at the 67LR receptor warrants further experimental verification, these collective findings underscore a critical methodological point: phenolic compounds like phenol red must be avoided in culture media when investigating the *in vitro* effects of polyphenols and Aβ at physiologically relevant, low concentrations.

#### 6.1.8. 67LR as a Redox Sensor

Although polyphenols and their metabolites physically bind to 67LR via their phenolic groups, their inherent redox sensitivity may also play a crucial role in cellular regulation. Previous studies have established that 67LR can function as a redox sensor [226]. Specifically, H_2_O_2_ induces the oxidation of redox-active cysteine thiols within the receptor; one of these cysteine residues is located directly within peptide G, while another lies in close proximity [226]. Additionally, the peptide G region features a Met–Trp–Trp–Met motif within its palindromic sequence. Because studies using artificial model proteins have demonstrated that methionine-flanked tryptophan residues can participate in electron transfer reactions [227], we postulate that this evolutionarily conserved motif in 67LR may support localized charge-transfer or redox interactions, particularly under oxidative conditions. Furthermore, 67LR has been reported to exhibit sulfhydryl oxidase-like activity [228]. It remains unclear whether transiently oxidized polyphenol species, such as semiquinone or quinone intermediates, interact with these redox-active vicinal sulfhydryls before being reduced back to their original phenolic states. Finally, EGCG binding to 67LR induces a modest, sublethal elevation in H_2_O_2_, a molecule increasingly recognized as an essential mediator of cellular signaling [229].

### 6.2. Neuroprotective Actions of Polyphenols by Inhibiting QR2

Resveratrol and quercetin inhibit QR2 with high affinity (Kd = 35 and 50 nM, respectively), and this inhibition leads to upregulation of antioxidant enzyme expression [230]. QR2 is overexpressed during aging and is particularly abundant in the hippocampus of patients with AD, where it likely contributes to metabolic stress and cognitive deficits [231,232]. Because specific QR2 inhibitors have been shown to reduce metabolic burden and reverse AD phenotypes in mouse models [231], inhibition of QR2 by polyphenols such as resveratrol and quercetin may improve cognitive function and offer therapeutic benefit in AD.

The exact physiological function of QR2 remains unclear; whether it primarily catalyzes quinone reduction or functions in cell signaling is still under investigation [233]. Unlike typical reductases, QR2 cannot use NAD(P)H as a reducing cofactor. Instead, it retains a functional FAD cofactor and cycles between oxidized and reduced states. These unique characteristics suggest that QR2 may act as a redox sensor and regulator rather than a traditional detoxifying enzyme for electrophilic quinones [233]. Finally, given the low concentrations of polyphenol aglycones achieved in the brain, it remains to be determined whether their more prevalent glucuronide and sulfate conjugates or their oxidative metabolites retain the ability to interact with and inhibit QR2 *in vivo*.

## 7. Indirect Neuroprotective Actions of Polyphenols – AD Prevention

### 7.1. Actions on Glial Cells in the CNS

The neuroprotective effects of polyphenols may also arise indirectly through the modulation of glial cell function. Aβ accumulation activates microglia and induces the production of proinflammatory cytokines, driving a chronic state of neuroinflammation [234]. Polyphenols effectively counter this cascade: resveratrol, for example, inhibits prostaglandin E2 (PGE2) production and free radical formation in activated microglia by modulating the cyclooxygenase/PGE2 signaling pathway [235]. Furthermore, resveratrol suppresses NF-κB transcriptional activation, thereby reducing cytokine secretion from both Aβ-activated astrocytes and microglia [236]. Similarly, EGCG exerts broad anti-inflammatory effects by transcriptionally downregulating cytokine release [237]. Notably, both resveratrol and EGCG activate SIRT1, which markedly attenuates Aβ-induced NF-κB signaling to confer strong neuroprotection [238]. Quercetin complements these actions by mitigating microglial inflammatory responses via activation of the Nrf2/heme oxygenase-1 pathway [239].

Despite these promising findings, most *in vitro* demonstrations of these anti-inflammatory effects have relied on micromolar concentrations of polyphenol aglycones. It remains to be determined whether the low nanomolar concentrations of their biologically relevant glucuronide and sulfate conjugates can elicit comparable responses. Additionally, while the PEDF peptide is known to mediate anti-inflammatory actions via 67LR in astrocytes [240], future studies must clarify whether polyphenols similarly leverage this receptor to exert their anti-inflammatory effects in glial cells.

### 7.2. Peripheral Actions

Although brain exposure to dietary quercetin, resveratrol, and EGCG is limited, these polyphenols may indirectly promote resilience to AD by attenuating systemic drivers of neurodegeneration through peripheral actions. Chronic elevations in circulating cytokines and sustained immune-cell activation are known to exacerbate cerebrovascular dysfunction and amplify neuroinflammation in AD [241,242]. *In vivo*, quercetin demonstrates robust systemic anti-inflammatory activity and improves vascular risk factors, such as blood pressure, which may translate into protection against cognitive decline [243,244]. Similarly, resveratrol ameliorates peripheral metabolic dysregulation (e.g., insulin resistance) via pathways involving SIRT1.

BBB integrity is compromised in AD, allowing blood-derived plasma proteins, such as fibrinogen, to extravasate into the brain parenchyma and promote neuronal degeneration [245–247]. EGCG has been shown to attenuate this serum extravasation by acting via 67LR [176]. Resveratrol also supports endothelial nitric oxide bioavailability and overall vascular function, which may indirectly protect the brain by improving cerebral perfusion and preserving BBB integrity [248,249]. Furthermore, *in vitro* studies report that micromolar concentrations of quercetin attenuate Aβ-induced cytotoxicity in human brain microvascular endothelial cells [250]. Because QR2 is expressed in endothelial cells and generates reactive ROS that can weaken the BBB [233], its inhibition by resveratrol and quercetin likely provides an additional mechanism for preserving barrier function. However, mechanistic evidence remains limited, and it is still unclear whether these protective effects are mediated *in vivo* by the parent aglycones or by their bioavailable glucuronide and sulfate conjugates acting through 67LR and QR2.

Furthermore, EGCG exerts significant gut-mediated systemic effects. Although extensively metabolized in the gut, EGCG can reshape the local microbiota and fortify intestinal barrier function. This fortification reduces the systemic translocation of pro-inflammatory microbial products, such as lipopolysaccharide (LPS), which are known to drive BBB impairment and neuroinflammatory signaling [251]. Collectively, these peripheral anti-inflammatory, metabolic, vasoprotective, and gut-restoring actions provide a cohesive mechanism by which quercetin, resveratrol, and EGCG can mitigate AD pathobiology, despite their low concentrations in the brain.

## 8. Combining Low-Dose Polyphenols for the Prevention of AD

When dietary polyphenols are consumed at standard, well-tolerated doses, only low nanomolar concentrations of the parent compounds and their conjugates reach the brain (Table 1). Crucially, these physiological concentrations fall below the *in vitro* binding affinities (*K*d) required to effectively engage 67LR. For example, consuming two cups of green tea yields an estimated brain EGCG concentration of just 10 nM [82], whereas its observed *K*d for 67LR is 40 nM [180]. Similarly, dietary quercetin-3-glucuronide reaches brain concentrations of approximately 1 nM [83], yet its binding affinity for the receptor's peptide G region is 4 nM [182]. Because our model indicates that the Aβ-PrP^c^ complex targets this exact region of 67LR, effective competition would require significantly higher brain concentrations than diet alone can provide. Consequently, at these low dietary levels, 67LR occupancy by polyphenols is likely suboptimal, yielding limited direct neuroprotection.

While escalating the dose of these polyphenols might intuitively seem like a strategy to enhance their efficacy for AD prevention, this approach carries a significant risk of peripheral toxicity. Although higher dosing may improve systemic exposure and CNS delivery, it is frequently accompanied by severe adverse effects. For instance, high-dose EGCG (or green tea extract) has been linked to hepatocellular injury in humans and produces marked hepatotoxicity in preclinical models [252]. Similarly, escalating the doses of resveratrol and quercetin has been associated with renal toxicity in animal studies [253,254]. and adverse drug interactions [255], respectively. Most concerningly, an unexpected finding from a randomized, placebo-controlled trial in patients with mild-to-moderate AD revealed that high-dose resveratrol administration was actually associated with greater brain volume loss on MRI compared to placebo [90].

An alternative strategy is to administer a combination of three or four well-characterized dietary polyphenols at low doses. Even if each compound achieves only low brain concentrations individually, agents converging on the same pathway (e.g., 67LR-mediated signaling) could produce additive effects that approximate the impact of a high-dose monotherapy. Moreover, because these polyphenols differ structurally, EGCG is a galloylated flavan-3-ol (catechin), quercetin circulates largely as glucuronide and sulfate conjugates, and resveratrol is a stilbene, they may engage complementary mechanisms that enable synergistic interactions. However, such combinations must also be carefully evaluated for potential receptor antagonism. Overall, supplementation with a limited set of structurally diverse, low-dose polyphenols represents a practical strategy to promote additive or synergistic neuroprotection for AD prevention while minimizing the risk of toxicity.

## 9. Conclusions and Perspectives

Given that AD develops over decades, its extended prodromal phase provides a realistic window for prevention. Because AD is multifactorial, however, meaningful risk reduction will likely require interventions that engage multiple, distinct pathways. Dietary polyphenols are highly appealing in this context because of their ability to influence several AD-relevant mechanisms simultaneously.

A central constraint in translating these benefits is pharmacokinetics. Most polyphenols are rapidly converted to glucuronide and sulfate conjugates and are extensively protein-bound, leaving only a small unbound fraction available for tissue distribution. Consequently, both parent aglycones and their conjugated metabolites typically reach the brain at only low nanomolar concentrations after limited passage across the BBB. Microbiota-derived metabolites follow a similar pattern: they are absorbed, rapidly conjugated, and circulate predominantly in these modified forms. Traditionally, these conjugates have been viewed merely as inactive excretory products or temporary reservoirs for regenerating aglycones. An important unresolved issue is whether these conjugated metabolites possess intrinsic biological activity, particularly through engagement of high-affinity targets, and therefore contribute directly to neuroprotection.

Although polyphenols can scavenge ROS, their direct antioxidant effects in vivo, especially in the brain, are likely negligible because of these low tissue concentrations and the generally weaker radical-scavenging capacity of conjugates compared with their parent aglycones. A more plausible mechanism is an “indirect antioxidant” action, whereby quercetin, resveratrol, EGCG, and their metabolites activate redox-responsive signaling pathways that upregulate endogenous cytoprotective enzymes. Such pathway-level reinforcement can persist even after the compounds themselves are cleared, providing a biologically credible route to sustained protection.

Two targets emphasized in this review are particularly relevant to AD pathogenesis and illustrate how ligands present at low concentrations can still produce meaningful effects. First, 67LR binds the AβO-PrP^c^ complex, promoting Aβ internalization and triggering downstream neurotoxic signaling. Quercetin, resveratrol, EGCG, and their conjugated metabolites bind this same 67LR site, competitively antagonizing AβO-PrP^c^ binding and activating neuroprotective cascades. Second, QR2 is an enzyme elevated in AD and associated with cognitive decline; resveratrol and quercetin bind QR2 with high affinity, inhibit its activity, and improve cognition in experimental settings. Notably, both 67LR and QR2 may function as redox sensors that react with oxidized polyphenol species, suggesting that redox-dependent chemistry contributes even within a receptor-based framework. Moving forward, it will be critical to determine whether other newly identified polyphenol receptors contain structural features that enable redox sensing, thereby clarifying when physical-binding targets and redox-sensing targets are distinct entities and when they are one and the same.

The ability of steroid hormones and catecholamines to act at low nanomolar or subnanomolar concentrations provides a highly relevant analogy: selective binding to a limited set of receptors enables substantial signal amplification through downstream transduction pathways. Polyphenols and their conjugates may similarly exert biological effects at low concentrations by engaging high-affinity targets that initiate defined signaling cascades. One illustrative mechanism is receptor-mediated elevation of cAMP, a second messenger whose amplification can drive broad transcriptional and functional responses despite minimal initial ligand exposure.

Ultimately, achieving therapeutically meaningful CNS exposure remains a major translational hurdle. Simply escalating doses to raise brain concentrations is not a viable solution, as higher intake increases the risk of peripheral toxicity, such as the hepatotoxicity reported with high EGCG exposure, raises the likelihood of adverse drug-nutrient interactions, and introduces safety concerns for long-term use.

A more practical strategy is low-dose combination supplementation using three or four well-characterized polyphenols. Even if each compound achieves only limited brain penetration individually, agents converging on the same protective node, such as 67LR-mediated signaling, could produce additive or synergistic effects that rival high-dose monotherapy while minimizing toxicity risks. Overall, defining which circulating conjugates and microbiota-derived metabolites are active, identifying their high-affinity targets, and delineating how redox-dependent processes intersect with receptor signaling will be essential for translating dietary polyphenols into an evidence-based clinical approach for AD prevention.

## Figures and Tables

**Table 1.** Distribution of quercetin, resveratrol, EGCG and their metabolites in plasma and brain

Parameters	Quercetin	Resveratrol	EGCG
Present in plants as	Glycoside form	Glycoside form	Aglycone
Absorption	Aglycone 20% [74]. Onion quercetin glucosides 60–80%[63]	75% [75]	Poorly absorbed [76]
Bioavailability	2% [74].	<1% [75]	0.1–0.3% [76]
Plasma concentration	Humans [64] Quercetin glycoside (equivalent to 100 mg aglycone) administered. Aglycone, N.D. Q-3-G, 500 nM Q-3-S, 300 nM Methylated-Q, 75 nM	Humans [77]: 25 mg *trans*-resveratrol Aglycone, <0.02 μM Conjugates all, 2 μM	Humans [78]: Catechin mixture containing 135 mg EGCG. Aglycone, 230 nM EGCG-4”-S, 170 nM EGCG-4”-G, 75 nM
Plasma protein binding	Humans [66]: Aglycone: 99%	Humans [67,79]: Aglycone: 98% Conjugates: 50%	Humans [68]: Aglycone: 90%
BBB permeability	Model: Rat endothelial/ glioma cell co-culture [80] Permeability coefficient 0.5 – 1.1 (10^−6^ cm/s) Transport ratio 2.1 to 2.5%	Model: Human brain microvascular endothelial cells [81] High passive permeability Transport ratio: >2%	Model: Co-cultures of endothelial cells, pericytes, astrocytes [82] Permeability coefficient 9.32 ± 0.32 (10^−6^ cm/s) Transport ratio: 2.82%
Brain concentration	Rats [83].: Red wine polyphenol mixture 150 mg was administered. Aglycone, N.D. Q-3-G, 1 nM	Rats [84]: Trans-resveratrol (15 mg/kg) by i.v. Aglycone, 0.17 nmol/g Mice [85]: Trans-resveratrol (150 mg/kg) by gavage R-3-G, 1.06 nmol/g R-3-S, 0.44 nmol/g	Rats [86]: EGCG 50 mg/kg administered. EGCG, 10 nM Rats [87]: High dose (500 mg/kg): EGCG, 0.5 nmol/g Mice [88]: EGCG, N.D.
CSF concentration	Sheep [89]: Powdered dry onion skin administered via intraluminal intubation Aglycone, 0.3 nM Conjugates, N.D.	Humans [90]: Escalating resveratrol doses (500 mg/day to 2g/day) were administered for 52 weeks. Aglycone, 2 nM R-3-G, 20 nM R-4’-G, 27 nM R-3-S, 39 nM	Humans [91]: MS patients were given 250 ml green tea (5.8 g tea leaves) Analyzed after 2 h. EGCG, N.D. (LOD: 10 nM)

*Note:* Treatments consisted of a single oral dose unless otherwise specified. Exceptions include human CSF measurements (daily dosing for 52 weeks) [90], resveratrol in rat brains (intravenous administration) [84], and powdered dry onion skin in sheep (intraluminal intubation) [89].

N.D., not detectable; Q-3-G, quercetin-3-glucuronide; Q-3-S, quercetin-3-sulfate; Q-7G/4′-S, quercetin-7-glucuronide-4′-sulfate; R-3-G, resveratrol-3-glucuronide; R-3-S, resveratrol-3-sulfate, LOD, limit of detection

**Table 2.** *Binding energies of polyphenol aglycones and their intestinal microbial metabolites to 37LRP, along with the predicted amino acids involved in ligand binding.* Three-dimensional structures of all compounds were generated using ChemDraw and converted to PDB format with Avogadro. Molecular docking was performed using the crystal structure of truncated 37LRP (PDB ID: 3BCH; amino acid residues 9–183) with AutoDock Vina (version 1.2.5) and AutoDockTools (version 1.5.7). Ligand-binding amino acid residues on 37LRP were identified using Discovery Studio (version 24.1). All glucuronide conjugates are primarily formed in the liver.

Polyphenol aglycones or their metabolites	Binding energy (kcal/mol)	Amino acids involved in the ligand binding to “A” site in 37LRP (a precursor protein for 67LR)
Quercetin	−8.34	Thr82, Arg85, Ala86, Lys89, Trp175, Trp176, Ala179 # (Phe90, Ala93, Ala168, His169, Gly172, Leu173)
Quercetin-3-glucuronide	−8.43	Arg85, Ala86, Lys89, Gly172, Trp175, Trp176 (Asn81, Thr82, Phe90, Ala93, His169, Leu173, Ala179)
*3,4-Dihydroxyphenylacetic acid	−5.06	Ala86, Lys89, Gly172 (Arg85, Phe90, Leu173, Trp175, Trp176, Ala179)
3,4-Dihydroxyphenylacetic acid-glucuronide	−7.11	Ala86, Ala168, His169, Leu173, Trp176 (Lys17, Ala20, Ala21, Arg85, Lys89, Gly172)
*trans*-Resveratrol	−6.65	Ala86, Lys89, His169, Gly172, Trp175, Trp176, Ala179 (Phe90, Ala93, Leu173)
Resveratrol-3-glucuronide	−7.78	Ala86, Lys89, His169, Trp175, Trp176, Ala 179 (Lys17, Ala20, Ala21, Phe90, Ala93, Gly172, Leu173)
*Dihydroresveratrol	−6.38	Ala86, Lys89, His169, Trp175, Trp176, Ala179 (Phe90, Ala93, Gly172, Leu173)
Dihydroresveratrol-3- glucuronide	−8.18	Thr82, Arg85, Ala86, Lys89, Trp175, Trp176, Ala179 (Asn81, Gln84, Phe90, Ala93, Ala168, Gly172)
Epigallocatechin-3-gallate	−9.31	Thr82, Arg85, Lys89, Gly172, Leu173, Trp176 (Lys17, Ala20, Ala21, Ala86, Phe90, Ala93, Ala168, His169, Trp175, Ala179)
Epigallocatechin-3-gallate- 4″-glucuronide	−9.96	Ala21, Thr82, Arg85, Lys89, Ala168, Leu173, Trp176 (Lys17, Ala20, Ala86, Phe90, Ala93, His169, Gly172, Trp175, Ala179, Arg180, Leu183)
*5-(3′,4′-Dihydroxyphenyl)- γ-valerolactone	−5.99	Ala86, Lys89, Trp175 (Arg85, Phe90, Gly172, Trp176, Ala179)
5-(3′,4′-Dihydroxyphenyl)-γ- valerolactone-3′-glucuronide	−6.84	Lys 89, Gly172, Trp176, Ala179 (Ala86, Phe90, Ala93, Leu173, Trp175, Leu183)

* Denotes intestinal microbial metabolites.

# Amino acid residues contributing to van der Waals interactions are indicated in parentheses.

## Data Availability

The raw data supporting information in Table 2 and Figure 2 in this article will be made available by the authors on request due to privacy.

## Abbreviations

The following abbreviations are used in this manuscript.

aMCI: Amnestic mild cognitive impairment
Aβ: Amyloid-β
AβO: Amyloid-β oligomers
AD: Alzheimer’s disease
BBB: Blood-brain barrier
BDNF: Brain-derived neurotrophic factor
CNS: Central nervous system
CREB: cAMP response element-binding protein
CSF: Cerebrospinal fluid
EGCG: (−)-Epigallocatechin-3-gallate
GAG: Glycosaminoglycan
67LR: 67-kDa laminin receptor
NMNAT: Nicotinamide mononucleotide adenylyltransferase
PDE: Phosphodiesterase
PKA: Protein kinase A
PP2A: Protein phosphatase 2A
PrP^C^: Cellular prion protein
Q-3-G: Quercetin-3-*O*-glucuronide
R-3-G: Resveratrol-3-*O*-glucuronide
ROS: Reactive oxygen species
SIRT1: Sirtuin 1 (NAD^+^-dependent deacetylase)

## References

[1] World Health Organization (WHO). Dementia (Fact sheet). Updated March 31, 2025.

[2] Estimation of the global prevalence of dementia in 2019 and forecasted prevalence in 2050: an analysis for the Global Burden of Disease Study 2019. Lancet Public Health 2022, 7, e105–e125, doi:10.1016/s2468-2667(21)00249-8. 34998485 PMC8810394

[3] Alzheimer's disease facts and figures. Alzheimer's & Dementia 2025, 21, e70235, doi:10.1002/alz.70235.

[4] Jack CR Jr.; Knopman DS; Jagust WJ; Petersen RC; Weiner MW; Aisen PS; Shaw LM; Vemuri P; Wiste HJ; Weigand SD, Tracking pathophysiological processes in Alzheimer's disease: an updated hypothetical model of dynamic biomarkers. Lancet Neurol 2013, 12, 207–216, doi:10.1016/s1474-4422(12)70291-0. 23332364 PMC3622225

[5] Tifratene K; Robert P; Metelkina A; Pradier C; Dartigues JF Progression of mild cognitive impairment to dementia due to AD in clinical settings. Neurology 2015, 85, 331–338, doi:doi:10.1212/WNL.0000000000001788. 26136516

[6] Rosenberg A; Mangialasche F; Ngandu T; Solomon A; Kivipelto M. Multidomain Interventions to Prevent Cognitive Impairment, Alzheimer's Disease, and Dementia: From FINGER to World-Wide FINGERS. J Prev Alzheimers Dis 2020, 7, 29–36, doi:10.14283/jpad.2019.41. 32010923 PMC7222931

[7] El Gaamouch F; Chen F; Ho L; Lin HY; Yuan C; Wong J; Wang J. Benefits of dietary polyphenols in Alzheimer's disease. Front Aging Neurosci 2022, 14, 1019942, doi:10.3389/fnagi.2022.1019942. PMC9792677 36583187

[8] Malar DS; Devi KP Dietary polyphenols for treatment of Alzheimer's disease--future research and development. Curr Pharm Biotechnol 2014, 15, 330–342, doi:10.2174/1389201015666140813122703. 25312617

[9] Bukhari SNA Dietary Polyphenols as Therapeutic Intervention for Alzheimer's Disease: A Mechanistic Insight. Antioxidants (Basel) 2022, 11, doi:10.3390/antiox11030554. 35326204 PMC8945272

[10] Kabir ER; Chowdhury NM; Yasmin H; Kabir MT; Akter R; Perveen A; Ashraf GM; Akter S; Rahman MH; Sweilam SH Unveiling the Potential of Polyphenols as Anti-Amyloid Molecules in Alzheimer's Disease. Curr Neuropharmacol 2023, 21, 787–807, doi:10.2174/1570159×20666221010113812. 36221865 PMC10227919

[11] Godos J; Micek A; Mena P; Del Rio D; Galvano F; Castellano S; Grosso G. Dietary (Poly)phenols and Cognitive Decline: A Systematic Review and Meta-Analysis of Observational Studies. Mol Nutr Food Res 2024, 68, e2300472, doi:10.1002/mnfr.202300472. 37888840

[12] Colizzi C. The protective effects of polyphenols on Alzheimer's disease: A systematic review. Alzheimers Dement (N Y) 2019, 5, 184–196, doi:10.1016/j.trci.2018.09.002. 31194101 PMC6551378

[13] Chen G; Su Y; Chen S; Lin T; Lin X. Polyphenols and Alzheimer's Disease: A Review on Molecular and Therapeutic Insights With In Silico Support. Food Sci Nutr 2025, 13, e70496, doi:10.1002/fsn3.70496. PMC12391731 40895150

[14] Wang X; Yang J; Zhang J; Yu G; Zhu J; Nie Y. Polyphenol consumption and neurodegeneration risk: a systematic meta-analysis of randomized controlled trials bridging nutrition and cognitive health. Food & Function 2026, https://doi.org/10.1039/D5FO05135E, doi:10.1039/D5FO05135E. 41524705

[15] Albadrani HM; Chauhan P; Ashique S; Babu MA; Iqbal D; Almutary AG; Abomughaid MM; Kamal M; Paiva-Santos AC; Alsaweed M, Mechanistic insights into the potential role of dietary polyphenols and their nanoformulation in the management of Alzheimer's disease. Biomed Pharmacother 2024, 174, 116376, doi:10.1016/j.biopha.2024.116376. 38508080

[16] Hasan S; Khatri N; Rahman ZN; Menezes AA; Martini J; Shehjar F; Mujeeb N; Shah ZA Neuroprotective Potential of Flavonoids in Brain Disorders. Brain Sci 2023, 13, doi:10.3390/brainsci13091258. 37759859 PMC10526484

[17] Hardy JA; Higgins GA Alzheimer's Disease: The Amyloid Cascade Hypothesis. Science 1992, 256, 184–185, doi:doi:10.1126/science.1566067. 1566067

[18] Selkoe DJ; Hardy J. The amyloid hypothesis of Alzheimer's disease at 25 years. EMBO Molecular Medicine 2016, 8, 595–608, doi:10.15252/emmm.201606210. 27025652 PMC4888851

[19] Hardy J; Selkoe DJ The Amyloid Hypothesis of Alzheimer's Disease: Progress and Problems on the Road to Therapeutics. Science 2002, 297, 353–356, doi:doi:10.1126/science.1072994. 12130773

[20] Shankar GM; Li S; Mehta TH; Garcia-Munoz A; Shepardson NE; Smith I; Brett FM; Farrell MA; Rowan MJ; Lemere CA, Amyloid-beta protein dimers isolated directly from Alzheimer's brains impair synaptic plasticity and memory. Nat Med 2008, 14, 837–842, doi:10.1038/nm1782. 18568035 PMC2772133

[21] Brody AH; Strittmatter SM Synaptotoxic Signaling by Amyloid Beta Oligomers in Alzheimer's Disease Through Prion Protein and mGluR5. Adv Pharmacol 2018, 82, 293–323, doi:10.1016/bs.apha.2017.09.007. 29413525 PMC5835229

[22] Petit D; Fernández SG; Zoltowska KM; Enzlein T; Ryan NS; O’Connor A; Szaruga M; Hill E; Vandenberghe R; Fox NC, Aβ profiles generated by Alzheimer’s disease causing PSEN1 variants determine the pathogenicity of the mutation and predict age at disease onset. Molecular Psychiatry 2022, 27, 2821–2832, doi:10.1038/s41380-022-01518-6. 35365805 PMC9156411

[23] Dyck C.H.v.; Swanson CJ; Aisen P; Bateman RJ; Chen C; Gee M; Kanekiyo M; Li D; Reyderman L; Cohen S, Lecanemab in Early Alzheimer’s Disease. New England Journal of Medicine 2023, 388, 9–21, doi:doi:10.1056/NEJMoa2212948. 36449413

[24] Gong CX; Dai CL; Liu F; Iqbal K. Multi-Targets: An Unconventional Drug Development Strategy for Alzheimer's Disease. Front Aging Neurosci 2022, 14, 837649, doi:10.3389/fnagi.2022.837649. PMC8864545 35222001

[25] Liu T; Rong Z; Li J; Wu H; Wei J. Three-dimensional interactive network: Mitochondrial-metabolic-calcium homeostasis driving Alzheimer’s disease. Genes & Diseases 2025, 10.1016/j.gendis.2025.101846, 101846, PMC12874427 41659206

[26] Zhang J; Zhang Y; Wang J; Xia Y; Zhang J; Chen L. Recent advances in Alzheimer’s disease: mechanisms, clinical trials and new drug development strategies. Signal Transduction and Targeted Therapy 2024, 9, 211, doi:10.1038/s41392-024-01911-3. 39174535 PMC11344989

[27] Mezzanotte M; Stanga S. Brain Iron Dyshomeostasis and Ferroptosis in Alzheimer's Disease Pathophysiology: Two Faces of the Same Coin. Aging Dis 2024, 16, 2615–2640, doi:10.14336/ad.2024.0094. 38913042 PMC12339084

[28] Klohs J. An Integrated View on Vascular Dysfunction in Alzheimer’s Disease. Neurodegenerative Diseases 2020, 19, 109–127, doi:10.1159/000505625. 32062666

[29] Arnsten AFT; Perone I; Wang M; Yang S; Uchendu S; Bolat D; Datta D. Dysregulated calcium signaling in the aged primate association cortices: vulnerability to Alzheimer's disease neuropathology. Front Aging Neurosci 2025, 17, 1610350, doi:10.3389/fnagi.2025.1610350. PMC12303958 40735190

[30] Cummings JL; Burstein AH; Fillit H. Alzheimer Combination Therapies: Overview and Scenarios. J Prev Alzheimers Dis 2025, 12, 100328, doi:10.1016/j.tjpad.2025.100328. PMC12627886 41145337

[31] Liu X; Dhana K; Barnes LL; Tangney CC; Agarwal P; Aggarwal N; Holland TM; Beck T; Evans DA; Rajan KB A healthy plant-based diet was associated with slower cognitive decline in African American older adults: a biracial community-based cohort. Am J Clin Nutr 2022, 116, 875–886, doi:10.1093/ajcn/nqac204. 35906190 PMC9535523

[32] Mani V; Arfeen M; Dhaked DK; Mohammed HA; Amirthalingam P; Elsisi HA Neuroprotective Effect of Methanolic Ajwa Seed Extract on Lipopolysaccharide-Induced Memory Dysfunction and Neuroinflammation: In Vivo, Molecular Docking and Dynamics Studies. Plants (Basel) 2023, 12, doi:10.3390/plants12040934. 36840284 PMC9964647

[33] Mani V; Parle M; Ramasamy K; Abdul Majeed AB Reversal of memory deficits by Coriandrum sativum leaves in mice. J Sci Food Agric 2011, 91, 186–192, doi:10.1002/jsfa.4171. 20848667

[34] Bao J; Liu W; Zhou HY; Gui YR; Yang YH; Wu MJ; Xiao YF; Shang JT; Long GF; Shu XJ Epigallocatechin-3-gallate Alleviates Cognitive Deficits in APP/PS1 Mice. Curr Med Sci 2020, 40, 18–27, doi:10.1007/s11596-020-2142-z. 32166661

[35] Morris MC; Tangney CC; Wang Y; Sacks FM; Barnes LL; Bennett DA; Aggarwal NT MIND diet slows cognitive decline with aging. Alzheimers Dement 2015, 11, 1015–1022, doi:10.1016/j.jalz.2015.04.011. 26086182 PMC4581900

[36] van den Brink AC; Brouwer-Brolsma EM; Berendsen AAM; van de Rest O. The Mediterranean, Dietary Approaches to Stop Hypertension (DASH), and Mediterranean-DASH Intervention for Neurodegenerative Delay (MIND) Diets Are Associated with Less Cognitive Decline and a Lower Risk of Alzheimer's Disease-A Review. Adv Nutr 2019, 10, 1040–1065, doi:10.1093/advances/nmz054. 31209456 PMC6855954

[37] Cherian L; Wang Y; Fakuda K; Leurgans S; Aggarwal N; Morris M. Mediterranean-Dash Intervention for Neurodegenerative Delay (MIND) Diet Slows Cognitive Decline After Stroke. J Prev Alzheimers Dis 2019, 6, 267–273, doi:10.14283/jpad.2019.28. 31686099 PMC7199507

[38] Huang L; Tao Y; Chen H; Chen X; Shen J; Zhao C; Xu X; He M; Zhu D; Zhang R, Mediterranean-Dietary Approaches to Stop Hypertension Intervention for Neurodegenerative Delay (MIND) Diet and Cognitive Function and its Decline: A Prospective Study and Meta-analysis of Cohort Studies. Am J Clin Nutr 2023, 118, 174–182, doi:10.1016/j.ajcnut.2023.04.025. 37105521

[39] Kuriyama S; Hozawa A; Ohmori K; Shimazu T; Matsui T; Ebihara S; Awata S; Nagatomi R; Arai H; Tsuji I. Green tea consumption and cognitive function: a cross-sectional study from the Tsurugaya Project 1. Am J Clin Nutr 2006, 83, 355–361, doi:10.1093/ajcn/83.2.355. 16469995

[40] Ide K; Yamada H; Takuma N; Park M; Wakamiya N; Nakase J; Ukawa Y; Sagesaka YM Green tea consumption affects cognitive dysfunction in the elderly: a pilot study. Nutrients 2014, 6, 4032–4042, doi:10.3390/nu6104032. 25268837 PMC4210905

[41] Singh N; Agrawal M; Doré S. Neuroprotective properties and mechanisms of resveratrol in in vitro and in vivo experimental cerebral stroke models. ACS Chem Neurosci 2013, 4, 1151–1162, doi:10.1021/cn400094w. 23758534 PMC3750679

[42] Rocha-González HI; Ambriz-Tututi M; Granados-Soto V. Resveratrol: a natural compound with pharmacological potential in neurodegenerative diseases. CNS Neurosci Ther 2008, 14, 234–247, doi:10.1111/j.1755-5949.2008.00045.x. 18684235 PMC6494027

[43] Pasinetti GM; Wang J; Ho L; Zhao W; Dubner L. Roles of resveratrol and other grape-derived polyphenols in Alzheimer's disease prevention and treatment. Biochim Biophys Acta 2015, 1852, 1202–1208, doi:10.1016/j.bbadis.2014.10.006. 25315300 PMC4380832

[44] Rege SD; Geetha T; Griffin GD; Broderick TL; Babu JR Neuroprotective effects of resveratrol in Alzheimer disease pathology. Front Aging Neurosci 2014, 6, 218, doi:10.3389/fnagi.2014.00218. 25309423 PMC4161050

[45] Arbo BD; André-Miral C; Nasre-Nasser RG; Schimith LE; Santos MG; Costa-Silva D; Muccillo-Baisch AL; Hort MA Resveratrol Derivatives as Potential Treatments for Alzheimer's and Parkinson's Disease. Front Aging Neurosci 2020, 12, 103, doi:10.3389/fnagi.2020.00103. 32362821 PMC7180342

[46] Lopez MS; Dempsey RJ; Vemuganti R. Resveratrol neuroprotection in stroke and traumatic CNS injury. Neurochem Int 2015, 89, 75–82, doi:10.1016/j.neuint.2015.08.009. 26277384 PMC4587342

[47] Paula PC; Angelica Maria SG; Luis CH; Gloria Patricia CG Preventive Effect of Quercetin in a Triple Transgenic Alzheimer's Disease Mice Model. Molecules 2019, 24, doi:10.3390/molecules24122287. 31226738 PMC6630340

[48] Sabogal-Guáqueta AM; Muñoz-Manco JI; Ramírez-Pineda JR; Lamprea-Rodriguez M; Osorio E; Cardona-Gómez GP The flavonoid quercetin ameliorates Alzheimer's disease pathology and protects cognitive and emotional function in aged triple transgenic Alzheimer's disease model mice. Neuropharmacology 2015, 93, 134–145, doi:10.1016/j.neuropharm.2015.01.027. 25666032 PMC4387064

[49] Moreno L; Puerta E; Suárez-Santiago JE; Santos-Magalhães NS; Ramirez MJ; Irache JM Effect of the oral administration of nanoencapsulated quercetin on a mouse model of Alzheimer's disease. Int J Pharm 2017, 517, 50–57, doi:10.1016/j.ijpharm.2016.11.061. 27915007

[50] Lv M; Yang S; Cai L; Qin LQ; Li BY; Wan Z. Effects of Quercetin Intervention on Cognition Function in APP/PS1 Mice was Affected by Vitamin D Status. Mol Nutr Food Res 2018, 62, e1800621, doi:10.1002/mnfr.201800621. 30328681

[51] Dzobo K; Hassen N; Senthebane DA; Thomford NE; Rowe A; Shipanga H; Wonkam A; Parker MI; Mowla S; Dandara C. Chemoresistance to Cancer Treatment: Benzo-α-Pyrene as Friend or Foe? Molecules 2018, 23, doi:10.3390/molecules23040930. 29673198 PMC6017867

[52] Zhang X; Hu J; Zhong L; Wang N; Yang L; Liu CC; Li H; Wang X; Zhou Y; Zhang Y, Quercetin stabilizes apolipoprotein E and reduces brain Aβ levels in amyloid model mice. Neuropharmacology 2016, 108, 179–192, doi:10.1016/j.neuropharm.2016.04.032. 27114256

[53] Zhu H; Huang J; Chen Y; Li X; Wen J; Tian M; Ren J; Zhou L; Yang Q. Resveratrol pretreatment protects neurons from oxygen–glucose deprivation/reoxygenation and ischemic injury through inhibiting ferroptosis. Bioscience, Biotechnology, and Biochemistry 2022, 86, 704–716, doi:10.1093/bbb/zbac048. 35357412

[54] Feng X; Liang N; Zhu D; Gao Q; Peng L; Dong H; Yue Q; Liu H; Bao L; Zhang J, Resveratrol inhibits β-amyloid-induced neuronal apoptosis through regulation of SIRT1-ROCK1 signaling pathway. PLoS One 2013, 8, e59888, doi:10.1371/journal.pone.0059888. PMC3610881 23555824

[55] Manczak M; Anekonda TS; Henson E; Park BS; Quinn J; Reddy PH Mitochondria are a direct site of A beta accumulation in Alzheimer's disease neurons: implications for free radical generation and oxidative damage in disease progression. Hum Mol Genet 2006, 15, 1437–1449. 16551656 10.1093/hmg/ddl066

[56] Raval AP; Dave KR; Pérez-Pinzón MA Resveratrol mimics ischemic preconditioning in the brain. J Cereb Blood Flow Metab 2006, 26, 1141–1147, doi:10.1038/sj.jcbfm.9600262. 16395277

[57] Rezai-Zadeh K; Shytle D; Sun N; Mori T; Hou H; Jeanniton D; Ehrhart J; Townsend K; Zeng J; Morgan D, Green tea epigallocatechin-3-gallate (EGCG) modulates amyloid precursor protein cleavage and reduces cerebral amyloidosis in Alzheimer transgenic mice. J Neurosci 2005, 25, 8807–8814, doi:10.1523/jneurosci.1521-05.2005. 16177050 PMC6725500

[58] Rezai-Zadeh K; Arendash GW; Hou H; Fernandez F; Jensen M; Runfeldt M; Shytle RD; Tan J. Green tea epigallocatechin-3-gallate (EGCG) reduces beta-amyloid mediated cognitive impairment and modulates tau pathology in Alzheimer transgenic mice. Brain Res 2008, 1214, 177–187, doi:10.1016/j.brainres.2008.02.107. 18457818

[59] Jia Y; Wang N; Liu X. Resveratrol and Amyloid-Beta: Mechanistic Insights. Nutrients 2017, 9, 1122. 29036903 10.3390/nu9101122 PMC5691738

[60] Sousa J.C.e.; Santana ACF; MagalhÃEs GJP Resveratrol in Alzheimer's disease: a review of pathophysiology and therapeutic potential. Arq Neuropsiquiatr 2020, 78, 501–511, doi:10.1590/0004-282X20200010. 32520230

[61] Zhang X-W; Chen J-Y; Ouyang D; Lu J-H Quercetin in Animal Models of Alzheimer’s Disease: A Systematic Review of Preclinical Studies. International Journal of Molecular Sciences 2020, 21, 493. 31941000 10.3390/ijms21020493 PMC7014205

[62] Cascella M; Bimonte S; Muzio MR; Schiavone V; Cuomo A. The efficacy of Epigallocatechin-3-gallate (green tea) in the treatment of Alzheimer's disease: an overview of pre-clinical studies and translational perspectives in clinical practice. Infect Agent Cancer 2017, 12, 36, doi:10.1186/s13027-017-0145-6. 28642806 PMC5477123

[63] Walle T; Otake Y; Walle UK; Wilson FA Quercetin glucosides are completely hydrolyzed in ileostomy patients before absorption. J Nutr 2000, 130, 2658–2661, doi:10.1093/jn/130.11.2658. 11053503

[64] Sudaka Y; Mitsui T; Kida H; Sultana MJ; Nishikawa M; Ikushiro S; Yamaguchi N. Validation of a quantitation method for conjugated quercetin in human plasma. Journal of Pharmaceutical and Biomedical Analysis 2025, 258, 116738, doi:10.1016/j.jpba.2025.116738. 39961223

[65] Vitaglione P; Sforza S; Galaverna G; Ghidini C; Caporaso N; Vescovi PP; Fogliano V; Marchelli R. Bioavailability of trans-resveratrol from red wine in humans. Mol Nutr Food Res 2005, 49, 495–504, doi:10.1002/mnfr.200500002. 15830336

[66] Boulton DW; Walle UK; Walle T. Extensive binding of the bioflavonoid quercetin to human plasma proteins. J Pharm Pharmacol 1998, 50, 243–249, doi:10.1111/j.2042-7158.1998.tb06183.x. 9530994

[67] Robinson K; Mock C; Liang D. Pre-formulation studies of resveratrol. Drug Dev Ind Pharm 2015, 41, 1464–1469, doi:10.3109/03639045.2014.958753. 25224342 PMC4427559

[68] Eaton JD; Williamson MP Multi-site binding of epigallocatechin gallate to human serum albumin measured by NMR and isothermal titration calorimetry. Biosci Rep 2017, 37, doi:10.1042/bsr20170209. PMC5484023 28424370

[69] Manach C; Scalbert A; Morand C; Rémésy C; Jiménez L. Polyphenols: food sources and bioavailability. The American Journal of Clinical Nutrition 2004, 79, 727–747, doi:10.1093/ajcn/79.5.727. 15113710

[70] Manach C; Williamson G; Morand C; Scalbert A; Rémésy C. Bioavailability and bioefficacy of polyphenols in humans. I. Review of 97 bioavailability studies. Am J Clin Nutr 2005, 81, 230s–242s, doi:10.1093/ajcn/81.1.230S. 15640486

[71] Crozier A; Del Rio D; Clifford MN Bioavailability of dietary flavonoids and phenolic compounds. Mol Aspects Med 2010, 31, 446–467, doi:10.1016/j.mam.2010.09.007. 20854839

[72] Del Rio D; Rodriguez-Mateos A; Spencer JP; Tognolini M; Borges G; Crozier A. Dietary (poly)phenolics in human health: structures, bioavailability, and evidence of protective effects against chronic diseases. Antioxid Redox Signal 2013, 18, 1818–1892, doi:10.1089/ars.2012.4581. 22794138 PMC3619154

[73] D'Archivio M; Filesi C; Varì R; Scazzocchio B; Masella R. Bioavailability of the polyphenols: status and controversies. Int J Mol Sci 2010, 11, 1321–1342, doi:10.3390/ijms11041321. 20480022 PMC2871118

[74] Xiong F; Zhang Y; Li T; Tang Y; Song S-Y; Zhou Q; Wang Y. A detailed overview of quercetin: implications for cell death and liver fibrosis mechanisms. Frontiers in Pharmacology 2024, Volume 15 - 2024, doi:10.3389/fphar.2024.1389179. PMC11157233 38855739

[75] Walle T. Bioavailability of resveratrol. Ann N Y Acad Sci 2011, 1215, 9–15, doi:10.1111/j.1749-6632.2010.05842.x. 21261636

[76] Pervin M; Unno K; Takagaki A; Isemura M; Nakamura Y. Function of Green Tea Catechins in the Brain: Epigallocatechin Gallate and its Metabolites. Int J Mol Sci 2019, 20, doi:10.3390/ijms20153630. 31349535 PMC6696481

[77] Walle T; Hsieh F; DeLegge MH; Oatis JE Jr.; Walle UK High absorption but very low bioavailability of oral resveratrol in humans. Drug Metab Dispos 2004, 32, 1377–1382, doi:10.1124/dmd.104.000885. 15333514

[78] Hayashi A; Terasaka S; Nukada Y; Kameyama A; Yamane M; Shioi R; Iwashita M; Hashizume K; Morita O. 4″-Sulfation Is the Major Metabolic Pathway of Epigallocatechin-3-gallate in Humans: Characterization of Metabolites, Enzymatic Analysis, and Pharmacokinetic Profiling. J Agric Food Chem 2022, 70, 8264–8273, doi:10.1021/acs.jafc.2c02150. 35786898 PMC9284555

[79] Burkon A; Somoza V. Quantification of free and protein-bound trans-resveratrol metabolites and identification of trans-resveratrol-C/O-conjugated diglucuronides - two novel resveratrol metabolites in human plasma. Mol Nutr Food Res 2008, 52, 549–557, doi:10.1002/mnfr.200700290. 18435437

[80] Youdim KA; Qaiser MZ; Begley DJ; Rice-Evans CA; Abbott NJ Flavonoid permeability across an in situ model of the blood–brain barrier. Free Radical Biology and Medicine 2004, 36, 592–604, doi:10.1016/j.freeradbiomed.2003.11.023. 14980703

[81] Alquisiras-Burgos I; González-Herrera IG; Alcalá-Alcalá S; Aguilera P. Nose-to Brain Delivery of Resveratrol, a Non-Invasive Method for the Treatment of Cerebral Ischemia. Drugs and Drug Candidates 2024, 3, 102–125.

[82] Pervin M; Unno K; Nakagawa A; Takahashi Y; Iguchi K; Yamamoto H; Hoshino M; Hara A; Takagaki A; Nanjo F, Blood brain barrier permeability of (−)-epigallocatechin gallate, its proliferation-enhancing activity of human neuroblastoma SH-SY5Y cells, and its preventive effect on age-related cognitive dysfunction in mice. Biochem Biophys Rep 2017, 9, 180–186, doi:10.1016/j.bbrep.2016.12.012. 28956003 PMC5614586

[83] Ho L; Ferruzzi MG; Janle EM; Wang J; Gong B; Chen TY; Lobo J; Cooper B; Wu QL; Talcott ST, Identification of brain-targeted bioactive dietary quercetin-3-O-glucuronide as a novel intervention for Alzheimer's disease. Faseb j 2013, 27, 769–781, doi:10.1096/fj.12-212118. 23097297 PMC3545533

[84] Juan ME; Maijó M; Planas JM Quantification of trans-resveratrol and its metabolites in rat plasma and tissues by HPLC. J Pharm Biomed Anal 2010, 51, 391–398, doi:10.1016/j.jpba.2009.03.026. 19406597

[85] Menet MC; Baron S; Taghi M; Diestra R; Dargère D; Laprévote O; Nivet-Antoine V; Beaudeux JL; Bédarida T; Cottart CH Distribution of trans-resveratrol and its metabolites after acute or sustained administration in mouse heart, brain, and liver. Mol Nutr Food Res 2017, 61, doi:10.1002/mnfr.201600686. 28160405

[86] Lin LC; Wang MN; Tseng TY; Sung JS; Tsai TH Pharmacokinetics of (−)-epigallocatechin-3-gallate in conscious and freely moving rats and its brain regional distribution. J Agric Food Chem 2007, 55, 1517–1524. 17256961 10.1021/jf062816a

[87] Nakagawa K; Miyazawa T. Absorption and distribution of tea catechin, (−)-epigallocatechin-3-gallate, in the rat. J Nutr Sci Vitaminol 1997, 43, 679–684. 9530620 10.3177/jnsv.43.679

[88] Lambert JD; Lee M-J; Lu H; Meng X; Hong JJJ; Seril DN; Sturgill MG; Yang CS Epigallocatechin-3-Gallate Is Absorbed but Extensively Glucuronidated Following Oral Administration to Mice. The Journal of Nutrition 2003, 133, 4172–4177. 14652367 10.1093/jn/133.12.4172

[89] Wiczkowski W; Skipor J; Misztal T; Szawara-Nowak D; Topolska J; Piskula MK Quercetin and isorhamnetin aglycones are the main metabolites of dietary quercetin in cerebrospinal fluid. Molecular Nutrition & Food Research 2015, 59, 1088–1094, doi:10.1002/mnfr.201400567. 25727325

[90] Turner RS; Thomas RG; Craft S; van Dyck CH; Mintzer J; Reynolds BA; Brewer JB; Rissman RA; Raman R; Aisen PS A randomized, double-blind, placebo-controlled trial of resveratrol for Alzheimer disease. Neurology 2015, 85, 1383–1391, doi:10.1212/wnl.0000000000002035. 26362286 PMC4626244

[91] Zini A; Del Rio D; Stewart AJ; Mandrioli J; Merelli E; Sola P; Nichelli P; Serafini M; Brighenti F; Edwards CA, Do flavan-3-ols from green tea reach the human brain? Nutr Neurosci 2006, 9, 57–61. 16910171 10.1080/10284150600637739

[92] Moon J-H; Tsushida T; Nakahara K; Terao J. Identification of quercetin 3-O-β-D-glucuronide as an antioxidative metabolite in rat plasma after oral administration of quercetin. Free Radical Biology and Medicine 2001, 30, 1274–1285, doi:10.1016/S0891-5849(01)00522-6. 11368925

[93] Tamura G; Gold C; Ferro-Luzzi A; Ames BN Fecalase: a model for activation of dietary glycosides to mutagens by intestinal flora. Proceedings of the National Academy of Sciences 1980, 77, 4961–4965, doi:doi:10.1073/pnas.77.8.4961. PMC349969 6933540

[94] Moon YJ; Wang L; DiCenzo R; Morris ME Quercetin pharmacokinetics in humans. Biopharm Drug Dispos 2008, 29, 205–217, doi:10.1002/bdd.605. 18241083

[95] Mullen W; Edwards CA; Crozier A. Absorption, excretion and metabolite profiling of methyl-, glucuronyl-, glucosyl- and sulpho-conjugates of quercetin in human plasma and urine after ingestion of onions. Br J Nutr 2006, 96, 107–116, doi:10.1079/bjn20061809. 16869998

[96] Walle T; Walle UK; Halushka PV Carbon dioxide is the major metabolite of quercetin in humans. J Nutr 2001, 131, 2648–2652, doi:10.1093/jn/131.10.2648. 11584085

[97] Baur JA; Sinclair DA Therapeutic potential of resveratrol: the in vivo evidence. Nat Rev Drug Discov 2006, 5, 493–506, doi:10.1038/nrd2060. 16732220

[98] Salehi B; Mishra AP; Nigam M; Sener B; Kilic M; Sharifi-Rad M; Fokou PVT; Martins N; Sharifi-Rad J. Resveratrol: A Double-Edged Sword in Health Benefits. Biomedicines 2018, 6, doi:10.3390/biomedicines6030091. 30205595 PMC6164842

[99] Bjelakovic G; Nikolova D; Gluud LL; Simonetti RG; Gluud C. Mortality in randomized trials of antioxidant supplements for primary and secondary prevention: systematic review and meta-analysis. Jama 2007, 297, 842–857. 17327526 10.1001/jama.297.8.842

[100] Almeida L; Vaz-da-Silva M; Falcão A; Soares E; Costa R; Loureiro AI; Fernandes-Lopes C; Rocha JF; Nunes T; Wright L, Pharmacokinetic and safety profile of trans-resveratrol in a rising multiple-dose study in healthy volunteers. Mol Nutr Food Res 2009, 53 Suppl 1, S7–15, doi:10.1002/mnfr.200800177. 19194969

[101] Cabrera C; Artacho R; Giménez R. Beneficial effects of green tea--a review. J Am Coll Nutr 2006, 25, 79–99, doi:10.1080/07315724.2006.10719518. 16582024

[102] Suganuma M; Okabe S; Oniyama M; Tada Y; Ito H; Fujiki H. Wide distribution of [3H](−)-epigallocatechin gallate, a cancer preventive tea polyphenol, in mouse tissue. Carcinogenesis 1998, 19, 1771–1776. 9806157 10.1093/carcin/19.10.1771

[103] Lee MJ; Maliakal P; Chen L; Meng X; Bondoc FY; Prabhu S; Lambert G; Mohr S; Yang CS Pharmacokinetics of tea catechins after ingestion of green tea and (−)-epigallocatechin-3-gallate by humans: formation of different metabolites and individual variability. Cancer Epidemiol Biomarkers Prev 2002, 11, 1025–1032. 12376503

[104] Capasso L; De Masi L; Sirignano C; Maresca V; Basile A; Nebbioso A; Rigano D; Bontempo P. Epigallocatechin Gallate (EGCG): Pharmacological Properties, Biological Activities and Therapeutic Potential. Molecules 2025, 30, doi:10.3390/molecules30030654. 39942757 PMC11821029

[105] Ishisaka A; Ichikawa S; Sakakibara H; Piskula MK; Nakamura T; Kato Y; Ito M; Miyamoto K; Tsuji A; Kawai Y, Accumulation of orally administered quercetin in brain tissue and its antioxidative effects in rats. Free Radic Biol Med 2011, 51, 1329–1336, doi:10.1016/j.freeradbiomed.2011.06.017. 21741473

[106] Huebbe P; Wagner AE; Boesch-Saadatmandi C; Sellmer F; Wolffram S; Rimbach G. Effect of dietary quercetin on brain quercetin levels and the expression of antioxidant and Alzheimer's disease relevant genes in mice. Pharmacol Res 2010, 61, 242–246, doi:10.1016/j.phrs.2009.08.006. 19720149

[107] Rahman MH; Akter R; Bhattacharya T; Abdel-Daim MM; Alkahtani S; Arafah MW; Al-Johani NS; Alhoshani NM; Alkeraishan N; Alhenaky A, Resveratrol and Neuroprotection: Impact and Its Therapeutic Potential in Alzheimer's Disease. Front Pharmacol 2020, 11, 619024, doi:10.3389/fphar.2020.619024. PMC7804889 33456444

[108] Shu XH; Wang LL; Li H; Song X; Shi S; Gu JY; Wu ML; Chen XY; Kong QY; Liu J. Diffusion Efficiency and Bioavailability of Resveratrol Administered to Rat Brain by Different Routes: Therapeutic Implications. Neurotherapeutics 2015, 12, 491–501, doi:10.1007/s13311-014-0334-6. 25588581 PMC4404447

[109] Yeh SL; Lin YC; Lin YL; Li CC; Chuang CH Comparing the metabolism of quercetin in rats, mice and gerbils. Eur J Nutr 2016, 55, 413–422, doi:10.1007/s00394-015-0862-9. 25691233

[110] Lambert JD; Lee MJ; Lu H; Meng X; Hong JJ; Seril DN; Sturgill MG; Yang CS Epigallocatechin-3-gallate is absorbed but extensively glucuronidated following oral administration to mice. J Nutr 2003, 133, 4172–4177, doi:10.1093/jn/133.12.4172. 14652367

[111] Kennedy DO Polyphenols and the human brain: plant “secondary metabolite” ecologic roles and endogenous signaling functions drive benefits. Adv Nutr 2014, 5, 515–533, doi:10.3945/an.114.006320. 25469384 PMC4188223

[112] Halliwell B; Rafter J; Jenner A. Health promotion by flavonoids, tocopherols, tocotrienols, and other phenols: direct or indirect effects? Antioxidant or not? American Journal of Clinical Nutrition 2005, 81, 268S–276S. 15640490 10.1093/ajcn/81.1.268S

[113] Schaffer S; Halliwell B. Do polyphenols enter the brain and does it matter? Some theoretical and practical considerations. Genes Nutr 2012, 7, 99–109, doi:10.1007/s12263-011-0255-5. 22012276 PMC3316747

[114] Sies H. Polyphenols and health: update and perspectives. Arch Biochem Biophys 2010, 501, 2–5, doi:10.1016/j.abb.2010.04.006. 20398620

[115] Williams RJ; Spencer JP; Rice-Evans C. Flavonoids: antioxidants or signalling molecules? Free Radic Biol Med 2004, 36, 838–849, doi:10.1016/j.freeradbiomed.2004.01.001. 15019969

[116] Justino GC; Santos MR; Canário S; Borges C; Florêncio MH; Mira L. Plasma quercetin metabolites: structure-antioxidant activity relationships. Arch Biochem Biophys 2004, 432, 109–121, doi:10.1016/j.abb.2004.09.007. 15519302

[117] Kode A; Rajendrasozhan S; Caito S; Yang SR; Megson IL; Rahman I. Resveratrol induces glutathione synthesis by activation of Nrf2 and protects against cigarette smoke-mediated oxidative stress in human lung epithelial cells. Am J Physiol Lung Cell Mol Physiol 2008, 294, L478–488, doi:10.1152/ajplung.00361.2007. 18162601

[118] Pullikotil P; Chen H; Muniyappa R; Greenberg CC; Yang S; Reiter CE; Lee JW; Chung JH; Quon MJ Epigallocatechin gallate induces expression of heme oxygenase-1 in endothelial cells via p38 MAPK and Nrf-2 that suppresses proinflammatory actions of TNF-α. J Nutr Biochem 2012, 23, 1134–1145, doi:10.1016/j.jnutbio.2011.06.007. 22137262 PMC3296830

[119] Tanigawa S; Fujii M; Hou DX Action of Nrf2 and Keap1 in ARE-mediated NQO1 expression by quercetin. Free Radic Biol Med 2007, 42, 1690–1703, doi:10.1016/j.freeradbiomed.2007.02.017. 17462537

[120] Dinkova-Kostova AT; Talalay P. Direct and indirect antioxidant properties of inducers of cytoprotective proteins. Mol Nutr Food Res 2008, 52 Suppl 1, S128–138, doi:10.1002/mnfr.200700195. 18327872

[121] Jung KA; Kwak MK The Nrf2 system as a potential target for the development of indirect antioxidants. Molecules 2010, 15, 7266–7291, doi:10.3390/molecules15107266. 20966874 PMC6259123

[122] Ahmad A; Syed FA; Singh S; Hadi SM Prooxidant activity of resveratrol in the presence of copper ions: mutagenicity in plasmid DNA. Toxicol Lett 2005, 159, 1–12, doi:10.1016/j.toxlet.2005.04.001. 15913925

[123] Tan J; Wang B; Zhu L. DNA binding and oxidative DNA damage induced by a quercetin copper(II) complex: potential mechanism of its antitumor properties. J Biol Inorg Chem 2009, 14, 727–739, doi:10.1007/s00775-009-0486-8. 19259707

[124] Plauth A; Geikowski A; Cichon S; Wowro SJ; Liedgens L; Rousseau M; Weidner C; Fuhr L; Kliem M; Jenkins G, Hormetic shifting of redox environment by pro-oxidative resveratrol protects cells against stress. Free Radic Biol Med 2016, 99, 608–622, doi:10.1016/j.freeradbiomed.2016.08.006. 27515816

[125] Elbling L; Herbacek I; Weiss RM; Jantschitsch C; Micksche M; Gerner C; Pangratz H; Grusch M; Knasmuller S; Berger W. Hydrogen peroxide mediates EGCG-induced antioxidant protection in human keratinocytes. Free Radic Biol Med 2010, 49, 1444–1452, doi:10.1016/j.freeradbiomed.2010.08.008. 20708679

[126] Ouyang J; Zhu K; Liu Z; Huang J. Prooxidant Effects of Epigallocatechin-3-Gallate in Health Benefits and Potential Adverse Effect. Oxid Med Cell Longev 2020, 2020, 9723686, doi:10.1155/2020/9723686. PMC7441425 32850004

[127] Forman HJ; Davies KJ; Ursini F. How do nutritional antioxidants really work: nucleophilic tone and para-hormesis versus free radical scavenging in vivo. Free Radic Biol Med 2014, 66, 24–35, doi:10.1016/j.freeradbiomed.2013.05.045. 23747930 PMC3852196

[128] Shah ZA; Li RC; Ahmad AS; Kensler TW; Yamamoto M; Biswal S; Doré S. The flavanol (−)-epicatechin prevents stroke damage through the Nrf2/HO1 pathway. J Cereb Blood Flow Metab 2010, 30, 1951–1961, doi:10.1038/jcbfm.2010.53. 20442725 PMC3002885

[129] Long LH; Clement MV; Halliwell B. Artifacts in cell culture: rapid generation of hydrogen peroxide on addition of (−)-epigallocatechin, (−)-epigallocatechin gallate, (+)-catechin, and quercetin to commonly used cell culture media. Biochemical & Biophysical Research Communications 2000, 273, 50–53. 10873562 10.1006/bbrc.2000.2895

[130] Halliwell B. Are polyphenols antioxidants or pro-oxidants? What do we learn from cell culture and in vivo studies? Arch Biochem Biophys 2008, 476, 107–112, doi:10.1016/j.abb.2008.01.028. 18284912

[131] Awad HM; Boersma MG; Vervoort J; Rietjens IM Peroxidase-catalyzed formation of quercetin quinone methide-glutathione adducts. Arch Biochem Biophys 2000, 378, 224–233, doi:10.1006/abbi.2000.1832. 10860540

[132] Steenwyk RC; Tan B. In vitro evidence for the formation of reactive intermediates of resveratrol in human liver microsomes. Xenobiotica 2010, 40, 62–71, doi:10.3109/00498250903337384. 19883238

[133] Sang S; Yang I; Buckley B; Ho CT; Yang CS Autoxidative quinone formation in vitro and metabolite formation in vivo from tea polyphenol (−)-epigallocatechin-3-gallate: studied by real-time mass spectrometry combined with tandem mass ion mapping. Free Radic Biol Med 2007, 43, 362–371, doi:10.1016/j.freeradbiomed.2007.04.008. 17602952 PMC2758168

[134] Lee J; Ebeler SE; Zweigenbaum JA; Mitchell AE UHPLC-(ESI)QTOF MS/MS profiling of quercetin metabolites in human plasma postconsumption of applesauce enriched with apple peel and onion. J Agric Food Chem 2012, 60, 8510–8520, doi:10.1021/jf302637t. 22867437

[135] Pervaiz S; Holme AL Resveratrol: its biologic targets and functional activity. Antioxid Redox Signal 2009, 11, 2851–2897, doi:10.1089/ars.2008.2412. 19432534

[136] Kim HS; Quon MJ; Kim JA New insights into the mechanisms of polyphenols beyond antioxidant properties; lessons from the green tea polyphenol, epigallocatechin 3-gallate. Redox Biol 2014, 2, 187–195, doi:10.1016/j.redox.2013.12.022. 24494192 PMC3909779

[137] Li H; Zhu F; Sun Y; Li B; Oi N; Chen H; Lubet RA; Bode AM; Dong Z. Select dietary phytochemicals function as inhibitors of COX-1 but not COX-2. PLoS One 2013, 8, e76452, doi:10.1371/journal.pone.0076452. PMC3789677 24098505

[138] Jiménez-Aliaga K; Bermejo-Bescós P; Benedí J; Martín-Aragón S. Quercetin and rutin exhibit antiamyloidogenic and fibril-disaggregating effects in vitro and potent antioxidant activity in APPswe cells. Life Sci 2011, 89, 939–945, doi:10.1016/j.lfs.2011.09.023. 22008478

[139] Feng Y; Wang XP; Yang SG; Wang YJ; Zhang X; Du XT; Sun XX; Zhao M; Huang L; Liu RT Resveratrol inhibits beta-amyloid oligomeric cytotoxicity but does not prevent oligomer formation. Neurotoxicology 2009, 30, 986–995, doi:10.1016/j.neuro.2009.08.013. 19744518

[140] Ehrnhoefer DE; Bieschke J; Boeddrich A; Herbst M; Masino L; Lurz R; Engemann S; Pastore A; Wanker EE EGCG redirects amyloidogenic polypeptides into unstructured, off-pathway oligomers. Nat Struct Mol Biol 2008, 15, 558–566, doi:10.1038/nsmb.1437. 18511942

[141] Kawabata K; Yoshioka Y; Terao J. Role of Intestinal Microbiota in the Bioavailability and Physiological Functions of Dietary Polyphenols. Molecules 2019, 24, doi:10.3390/molecules24020370. 30669635 PMC6359708

[142] Peng X; Zhang Z; Zhang N; Liu L; Li S; Wei H. In vitro catabolism of quercetin by human fecal bacteria and the antioxidant capacity of its catabolites. Food Nutr Res 2014, 58, doi:10.3402/fnr.v58.23406. PMC3991839 24765061

[143] Kasahara K; Kerby RL; Aquino-Martinez R; Evered AH; Cross T-WL; Everhart J; Ulland TK; Kay CD; Bolling BW; Bäckhed F, Gut microbes modulate the effects of the flavonoid quercetin on atherosclerosis. npj Biofilms and Microbiomes 2025, 11, 12, doi:10.1038/s41522-024-00626-1. 39794320 PMC11723976

[144] Bode LM; Bunzel D; Huch M; Cho GS; Ruhland D; Bunzel M; Bub A; Franz CM; Kulling SE In vivo and in vitro metabolism of trans-resveratrol by human gut microbiota. Am J Clin Nutr 2013, 97, 295–309, doi:10.3945/ajcn.112.049379. 23283496

[145] Iglesias-Aguirre CE; Vallejo F; Beltrán D; Aguilar-Aguilar E; Puigcerver J; Alajarín M; Berná J; Selma MV; Espín JC Lunularin Producers versus Non-producers: Novel Human Metabotypes Associated with the Metabolism of Resveratrol by the Gut Microbiota. J Agric Food Chem 2022, 70, 10521–10531, doi:10.1021/acs.jafc.2c04518. 35981285 PMC9449969

[146] Takagaki A; Nanjo F. Metabolism of (−)-epigallocatechin gallate by rat intestinal flora. J Agric Food Chem 2010, 58, 1313–1321, doi:10.1021/jf903375s. 20043675

[147] Roowi S; Stalmach A; Mullen W; Lean ME; Edwards CA; Crozier A. Green tea flavan-3-ols: colonic degradation and urinary excretion of catabolites by humans. J Agric Food Chem 2010, 58, 1296–1304, doi:10.1021/jf9032975. 20041649

[148] Liu Z; de Bruijn WJC; Bruins ME; Vincken JP Reciprocal Interactions between Epigallocatechin-3-gallate (EGCG) and Human Gut Microbiota In Vitro. J Agric Food Chem 2020, 68, 9804–9815, doi:10.1021/acs.jafc.0c03587. 32808768 PMC7496747

[149] Liu C; Boeren S; Miro Estruch I; Rietjens I. The Gut Microbial Metabolite Pyrogallol Is a More Potent Inducer of Nrf2-Associated Gene Expression Than Its Parent Compound Green Tea (−)-Epigallocatechin Gallate. Nutrients 2022, 14, doi:10.3390/nu14163392. 36014899 PMC9414524

[150] Lasa A; Churruca I; Eseberri I; Andrés-Lacueva C; Portillo MP Delipidating effect of resveratrol metabolites in 3T3-L1 adipocytes. Mol Nutr Food Res 2012, 56, 1559–1568, doi:10.1002/mnfr.201100772. 22945685

[151] Klimas R; Mikus G. Morphine-6-glucuronide is responsible for the analgesic effect after morphine administration: a quantitative review of morphine, morphine-6-glucuronide, and morphine-3-glucuronide. Br J Anaesth 2014, 113, 935–944, doi:10.1093/bja/aeu186. 24985077

[152] Buhl AE; Waldon DJ; Baker CA; Johnson GA Minoxidil sulfate is the active metabolite that stimulates hair follicles. J Invest Dermatol 1990, 95, 553–557, doi:10.1111/1523-1747.ep12504905. 2230218

[153] Bourasset F; Cisternino S; Temsamani J; Scherrmann JM Evidence for an active transport of morphine-6-beta-d-glucuronide but not P-glycoprotein-mediated at the blood-brain barrier. J Neurochem 2003, 86, 1564–1567, doi:10.1046/j.1471-4159.2003.01990.x. 12950465

[154] Yamada H; Ishii K; Ishii Y; Ieiri I; Nishio S; Morioka T; Oguri K. Formation of highly analgesic morphine-6-glucuronide following physiologic concentration of morphine in human brain. J Toxicol Sci 2003, 28, 395–401, doi:10.2131/jts.28.395. 14746343

[155] Abdullahi W; Davis TP; Ronaldson PT Functional Expression of P-glycoprotein and Organic Anion Transporting Polypeptides at the Blood-Brain Barrier: Understanding Transport Mechanisms for Improved CNS Drug Delivery? Aaps j 2017, 19, 931–939, doi:10.1208/s12248-017-0081-9. 28447295 PMC5505261

[156] Ishisaka A; Mukai R; Terao J; Shibata N; Kawai Y. Specific localization of quercetin-3-O-glucuronide in human brain. Arch Biochem Biophys 2014, 557, 11–17, doi:10.1016/j.abb.2014.05.025. 24893148

[157] Fernández-Castillejo S; Macià A; Motilva MJ; Catalán Ú; Solà R. Endothelial Cells Deconjugate Resveratrol Metabolites to Free Resveratrol: A Possible Role in Tissue Factor Modulation. Mol Nutr Food Res 2019, 63, e1800715, doi:10.1002/mnfr.201800715. 30570816

[158] Ouzzine M; Gulberti S; Ramalanjaona N; Magdalou J; Fournel-Gigleux S.f. The UDP-glucuronosyltransferases of the blood-brain barrier: their role in drug metabolism and detoxication. Front Cell Neurosci 2014, 8, 349, doi:10.3389/fncel.2014.00349. 25389387 PMC4211562

[159] Sabolovic N; Heurtaux T; Humbert AC; Krisa S; Magdalou J. cis- and trans-Resveratrol are glucuronidated in rat brain, olfactory mucosa and cultured astrocytes. Pharmacology 2007, 80, 185–192, doi:10.1159/000104149. 17579296

[160] Patel KR; Andreadi C; Britton RG; Horner-Glister E; Karmokar A; Sale S; Brown VA; Brenner DE; Singh R; Steward WP, Sulfate metabolites provide an intracellular pool for resveratrol generation and induce autophagy with senescence. Sci Transl Med 2013, 5, 205ra133, doi:10.1126/scitranslmed.3005870. 24089405

[161] Menendez C; Dueñas M; Galindo P; González-Manzano S; Jimenez R; Moreno L; Zarzuelo MJ; Rodríguez-Gómez I; Duarte J; Santos-Buelga C, Vascular deconjugation of quercetin glucuronide: the flavonoid paradox revealed? Mol Nutr Food Res 2011, 55, 1780–1790, doi:10.1002/mnfr.201100378. 22144045

[162] Shi Q; Haenen GR; Maas L; Arlt VM; Spina D; Vasquez YR; Moonen E; Veith C; Van Schooten FJ; Godschalk RWL Inflammation-associated extracellular β-glucuronidase alters cellular responses to the chemical carcinogen benzo[a]pyrene. Arch Toxicol 2016, 90, 2261–2273, doi:10.1007/s00204-015-1593-7. 26438400 PMC4982897

[163] El Masri R; Crétinon Y; Gout E; Vivès RR HS and Inflammation: A Potential Playground for the Sulfs? Frontiers in Immunology 2020, Volume 11 - 2020, doi:10.3389/fimmu.2020.00570. PMC7147386 32318065

[164] Rao NC; Barsky SH; Terranova VP; Liotta LA Isolation of a tumor cell laminin receptor. Biochem Biophys Res Commun 1983, 111, 804–808, doi:10.1016/0006-291x(83)91370-0. 6301485

[165] Lesot H; Kühl U; Mark K. Isolation of a laminin-binding protein from muscle cell membranes. Embo j 1983, 2, 861–865, doi:10.1002/j.1460-2075.1983.tb01514.x. 16453457 PMC555201

[166] Malinoff HL; Wicha MS Isolation of a cell surface receptor protein for laminin from murine fibrosarcoma cells. J Cell Biol 1983, 96, 1475–1479, doi:10.1083/jcb.96.5.1475. 6302102 PMC2112662

[167] DiGiacomo V; Meruelo D. Looking into laminin receptor: critical discussion regarding the non-integrin 37/67-kDa laminin receptor/RPSA protein. Biol Rev Camb Philos Soc 2016, 91, 288–310. 25630983 10.1111/brv.12170 PMC5249262

[168] Nelson J; McFerran NV; Pivato G; Chambers E; Doherty C; Steele D; Timson DJ The 67 kDa laminin receptor: structure, function and role in disease. Bioscience Reports 2008, 28, 33–48. 18269348 10.1042/BSR20070004

[169] Rea VE; Rossi FW; De Paulis A; Ragno P; Selleri C; Montuori N. 67 kDa laminin receptor: structure, function and role in cancer and infection. Infez Med 2012, 20 Suppl 2, 8–12. 23042000

[170] Ardini E; Pesole G; Tagliabue E; Magnifico A; Castronovo V; Sobel ME; Colnaghi MI; Ménard S. The 67-kDa laminin receptor originated from a ribosomal protein that acquired a dual function during evolution. Mol Biol Evol 1998, 15, 1017–1025, doi:10.1093/oxfordjournals.molbev.a026000. 9718729

[171] Pinnock EC; Jovanovic K; Pinto MG; Ferreira E; Dias BDC; Penny C; Knackmuss S; Reusch U; Little M; Schatzl HM, LRP/LR Antibody Mediated Rescuing of Amyloid-β-Induced Cytotoxicity is Dependent on PrPc in Alzheimer’s Disease. Journal of Alzheimer’s Disease 2016, 49, 645–657, doi:10.3233/jad-150482. 26484914

[172] Da Costa Dias B; Jovanovic K; Gonsalves D; Moodley K; Reusch U; Knackmuss S; Weinberg MS; Little M; Weiss SF The 37kDa/67kDa laminin receptor acts as a receptor for Abeta42 internalization. Sci Rep 2014, 4, 5556, doi:10.1038/srep05556. 24990253 PMC4080222

[173] Liu M; Li N; Guo W; Jia L; Jiang H; Li Z; Wang J; Zhang X; Zhu R; Bao C, RPSA distribution and expression in tissues and immune cells of pathogen-infected mice. Microb Pathog 2021, 152, 104609, doi:10.1016/j.micpath.2020.104609. 33217534

[174] Gundimeda U; McNeill TH; Schiffman JE; Hinton DR; Gopalakrishna R. Green tea polyphenols potentiate the action of nerve growth factor to induce neuritogenesis: possible role of reactive oxygen species. Journal of Neuroscience Research 2010, 88, 3644–3655. 20936703 10.1002/jnr.22519 PMC2965808

[175] Gundimeda U; McNeill TH; Fan TK; Deng R; Rayudu D; Chen Z; Cadenas E; Gopalakrishna R. Green tea catechins potentiate the neuritogenic action of brain-derived neurotrophic factor: role of 67-kDa laminin receptor and hydrogen peroxide. Biochem Biophys Res Commun 2014, 445, 218–224, doi:10.1016/j.bbrc.2014.01.166. 24508265

[176] Kim JE; Park H; Jeong MJ; Kang TC Epigallocatechin-3-Gallate and PEDF 335 Peptide, 67LR Activators, Attenuate Vasogenic Edema, and Astroglial Degeneration Following Status Epilepticus. Antioxidants (Basel) 2020, 9, doi:10.3390/antiox9090854. 32933011 PMC7555521

[177] Liang J; Luo Q; Shen N; Qin X; Jia C; Chao Z; Zhang L; Qin H; Liu X; Quan X, PEDF Protects Endothelial Barrier Integrity during Acute Myocardial Infarction via 67LR. Int J Mol Sci 2023, 24, doi:10.3390/ijms24032787. 36769107 PMC9917376

[178] Mrówczyńska E; Machalica K; Mazur AJ Non-integrin laminin receptor (LamR) plays a role in axonal outgrowth from chicken DRG via modulating the Akt and Erk signaling. Frontiers in Cell and Developmental Biology 2024, Volume 12 - 2024, doi:10.3389/fcell.2024.1433947. PMC11322362 39144252

[179] Blazejewski SM; Bennison SA; Ha NT; Liu X; Smith TH; Dougherty KJ; Toyo-Oka K. Rpsa Signaling Regulates Cortical Neuronal Morphogenesis via Its Ligand, PEDF, and Plasma Membrane Interaction Partner, Itga6. Cereb Cortex 2022, 32, 770–795, doi:10.1093/cercor/bhab242. 34347028 PMC8841558

[180] Tachibana H; Koga K; Fujimura Y; Yamada K. A receptor for green tea polyphenol EGCG. Nature Structural & Molecular Biology 2004, 11, 380–381. 10.1038/nsmb743 15024383

[181] Fujimura Y; Sumida M; Sugihara K; Tsukamoto S; Yamada K; Tachibana H. Green Tea Polyphenol EGCG Sensing Motif on the 67-kDa Laminin Receptor. PLoS ONE [Electronic Resource] 2012, 7. 10.1371/journal.pone.0037942 PMC3362541 22666419

[182] Gopalakrishna R; Oh A; Hou L; Lee E; Aguilar J; Li A; Mack WJ Flavonoid quercetin and its glucuronide and sulfate conjugates bind to 67-kDa laminin receptor and prevent neuronal cell death induced by serum starvation. Biochem Biophys Res Commun 2023, 671, 116–123, doi:10.1016/j.bbrc.2023.06.007. 37300941 PMC10527810

[183] Gopalakrishna R; Aguilar J; Oh A; Lee E; Hou L; Lee T; Xu E; Nguyen J; Mack WJ Resveratrol and its metabolites elicit neuroprotection via high-affinity binding to the laminin receptor at low nanomolar concentrations. FEBS Lett 2024, 598, 995–1007, doi:10.1002/1873-3468.14835. 38413095 PMC11087200

[184] Gopalakrishna R; Aguilar J; Lee E; Mack WJ Neuroprotection by resveratrol-glucuronide and quercetin-glucuronide via binding to polyphenol- and glycosaminoglycan-binding sites in the laminin receptor. Neural Regeneration Research 2025, 20. 10.4103/NRR.NRR-D-24-00160 PMC11433912 38886954

[185] Hayashi D; Mouchlis VD; Okamoto S; Namba T; Wang L; Li S; Ueda S; Yamanoue M; Tachibana H; Arai H, Vitamin E functions by association with a novel binding site on the 67 kDa laminin receptor activating diacylglycerol kinase. J Nutr Biochem 2022, 110, 109129, doi:10.1016/j.jnutbio.2022.109129. PMC10243646 35977663

[186] Namba T, H D, Fukuda I, Ueda S, and Shirai Y. . Tocotrienols activate diacylglycerol kinase a via 67 KDa laminin receptor. Functional Food Science 2025, 5, 160–169.

[187] Prakash S; Ghosh A; Nayek A; Kiran S. The Platelet Aggregation Inhibition Activity of Polyphenols can be Mediated by 67kda Laminin Receptor: A New Therapeutic Strategy For the Treatment of Venous Thromboembolism. Cardiovasc Hematol Agents Med Chem 2023, 22, 1–6, doi:10.2174/1871525721666230228120500. 36852811

[188] Bae J; Kumazoe M; Murata K; Fujimura Y; Tachibana H. Procyanidin C1 Inhibits Melanoma Cell Growth by Activating 67-kDa Laminin Receptor Signaling. Mol Nutr Food Res 2020, 64, e1900986, doi:10.1002/mnfr.201900986. 32103628

[189] Liu Z; Hu M. Natural polyphenol disposition via coupled metabolic pathways. Expert Opin Drug Metab Toxicol 2007, 3, 389–406, doi:10.1517/17425255.3.3.389. 17539746 PMC2777985

[190] Järvinen E; Deng F; Kiander W; Sinokki A; Kidron H; Sjöstedt N. The Role of Uptake and Efflux Transporters in the Disposition of Glucuronide and Sulfate Conjugates. Frontiers in Pharmacology 2022, 12 – 2021, doi:10.3389/fphar.2021.802539. PMC8793843 35095509

[191] Hundt C; Peyrin JM; Haïk S; Gauczynski S; Leucht C; Rieger R; Riley ML; Deslys JP; Dormont D; Lasmézas CI, Identification of interaction domains of the prion protein with its 37-kDa/67-kDa laminin receptor. Embo j 2001, 20, 5876–5886, doi:10.1093/emboj/20.21.5876. 11689428 PMC125289

[192] Cardin AD; Weintraub HJ Molecular modeling of protein-glycosaminoglycan interactions. Arteriosclerosis: An Official Journal of the American Heart Association, Inc. 1989, 9, 21–32, doi:doi:10.1161/01.ATV.9.1.21. 2463827

[193] Crijns H; Adyns L; Ganseman E; Cambier S; Vandekerckhove E; Pörtner N; Vanbrabant L; Struyf S; Gerlza T; Kungl A, Affinity and Specificity for Binding to Glycosaminoglycans Can Be Tuned by Adapting Peptide Length and Sequence. Int J Mol Sci 2021, 23, doi:10.3390/ijms23010447. 35008874 PMC8745253

[194] Jarosz-Griffiths HH; Noble E; Rushworth JV; Hooper NM Amyloid-beta Receptors: The Good, the Bad, and the Prion Protein. J Biol Chem 2016, 291, 3174–3183, doi:10.1074/jbc.R115.702704. 26719327 PMC4751366

[195] Mroczko B; Groblewska M; Litman-Zawadzka A; Kornhuber J; Lewczuk P. Cellular Receptors of Amyloid β Oligomers (AβOs) in Alzheimer's Disease. Int J Mol Sci 2018, 19, doi:10.3390/ijms19071884. 29954063 PMC6073792

[196] Laurén J; Gimbel DA; Nygaard HB; Gilbert JW; Strittmatter SM Cellular prion protein mediates impairment of synaptic plasticity by amyloid-β oligomers. Nature 2009, 457, 1128–1132, doi:10.1038/nature07761. 19242475 PMC2748841

[197] Smith LM; Kostylev MA; Lee S; Strittmatter SM Systematic and standardized comparison of reported amyloid-β receptors for sufficiency, affinity, and Alzheimer's disease relevance. J Biol Chem 2019, 294, 6042–6053, doi:10.1074/jbc.RA118.006252. 30787106 PMC6463724

[198] Ferreira E; Bignoux MJ; Otgaar TC; Tagliatti N; Jovanovic K; Letsolo BT; Weiss SFT LRP/LR specific antibody IgG1-iS18 impedes neurodegeneration in Alzheimer's disease mice. Oncotarget 2018, 9, 27059–27073, doi:10.18632/oncotarget.25473. 29930750 PMC6007457

[199] Gopalakrishna R; Lin CY; Oh A; Le C; Yang S; Hicks A; Kindy MS; Mack WJ; Bhat NR cAMP-induced decrease in cell-surface laminin receptor and cellular prion protein attenuates amyloid-β uptake and amyloid-β-induced neuronal cell death. FEBS Lett 2022, 596, 2914–2927, doi:10.1002/1873-3468.14467. 35971617 PMC9712173

[200] Park SJ; Ahmad F; Philp A; Baar K; Williams T; Luo H; Ke H; Rehmann H; Taussig R; Brown AL, Resveratrol ameliorates aging-related metabolic phenotypes by inhibiting cAMP phosphodiesterases. Cell 2012, 148, 421–433, doi:10.1016/j.cell.2012.01.017. 22304913 PMC3431801

[201] Kelly MP Cyclic nucleotide signaling changes associated with normal aging and age-related diseases of the brain. Cell Signal 2018, 42, 281–291, doi:10.1016/j.cellsig.2017.11.004. 29175000 PMC5732030

[202] Onoue S; Endo K; Ohshima K; Yajima T; Kashimoto K. The neuropeptide PACAP attenuates beta-amyloid (1–42)-induced toxicity in PC12 cells. Peptides 2002, 23, 1471–1478, doi:10.1016/s0196-9781(02)00085-2. 12182949

[203] Han P; Caselli RJ; Baxter L; Serrano G; Yin J; Beach TG; Reiman EM; Shi J. Association of pituitary adenylate cyclase-activating polypeptide with cognitive decline in mild cognitive impairment due to Alzheimer disease. JAMA Neurol 2015, 72, 333–339, doi:10.1001/jamaneurol.2014.3625. 25599520 PMC5924703

[204] Rat D; Schmitt U; Tippmann F; Dewachter I; Theunis C; Wieczerzak E; Postina R; van Leuven F; Fahrenholz F; Kojro E. Neuropeptide pituitary adenylate cyclase-activating polypeptide (PACAP) slows down Alzheimer's disease-like pathology in amyloid precursor protein-transgenic mice. Faseb j 2011, 25, 3208–3218, doi:10.1096/fj.10-180133. 21593432 PMC3157688

[205] Sanders O; Rajagopal L. Phosphodiesterase Inhibitors for Alzheimer's Disease: A Systematic Review of Clinical Trials and Epidemiology with a Mechanistic Rationale. J Alzheimers Dis Rep 2020, 4, 185–215, doi:10.3233/adr-200191. 32715279 PMC7369141

[206] Song XJ; Wang ZB; Gan Q; Walters ET cAMP and cGMP contribute to sensory neuron hyperexcitability and hyperalgesia in rats with dorsal root ganglia compression. J Neurophysiol 2006, 95, 479–492, doi:10.1152/jn.00503.2005. 16120663

[207] Amidfar M; de Oliveira J; Kucharska E; Budni J; Kim Y-K The role of CREB and BDNF in neurobiology and treatment of Alzheimer's disease. Life Sciences 2020, 257, 118020, doi:10.1016/j.lfs.2020.118020. 32603820

[208] Sakamoto K; Karelina K; Obrietan K. CREB: a multifaceted regulator of neuronal plasticity and protection. J Neurochem 2011, 116, 1–9, doi:10.1111/j.1471-4159.2010.07080.x. 21044077 PMC3575743

[209] Saura CA; Valero J. The role of CREB signaling in Alzheimer's disease and other cognitive disorders. Rev Neurosci 2011, 22, 153–169, doi:10.1515/rns.2011.018. 21476939

[210] Gerhart-Hines Z; Dominy JE Jr.; Blättler SM; Jedrychowski MP; Banks AS; Lim JH; Chim H; Gygi SP; Puigserver P. The cAMP/PKA pathway rapidly activates SIRT1 to promote fatty acid oxidation independently of changes in NAD(+). Mol Cell 2011, 44, 851–863, doi:10.1016/j.molcel.2011.12.005. 22195961 PMC3331675

[211] Mitani T; Watanabe S; Wada K; Fujii H; Nakamura S; Katayama S. Intracellular cAMP contents regulate NAMPT expression via induction of C/EBPβ in adipocytes. Biochemical and Biophysical Research Communications 2020, 522, 770–775, doi:10.1016/j.bbrc.2019.11.165. 31791580

[212] Wu S.-k.; Wang L; Wang F; Zhang J. Resveratrol improved mitochondrial biogenesis by activating SIRT1/PGC-1α signal pathway in SAP. Scientific Reports 2024, 14, 26216, doi:10.1038/s41598-024-76825-9. 39482340 PMC11528064

[213] Guan G; Chen Y; Dong Y. Unraveling the AMPK-SIRT1-FOXO Pathway: The In-Depth Analysis and Breakthrough Prospects of Oxidative Stress-Induced Diseases. Antioxidants (Basel) 2025, 14, doi:10.3390/antiox14010070. 39857404 PMC11763278

[214] Jiao F; Gong Z. The Beneficial Roles of SIRT1 in Neuroinflammation-Related Diseases. Oxid Med Cell Longev 2020, 2020, 6782872, doi:10.1155/2020/6782872. PMC7519200 33014276

[215] Ma X; Sun Z; Han X; Li S; Jiang X; Chen S; Zhang J; Lu H. Neuroprotective Effect of Resveratrol via Activation of Sirt1 Signaling in a Rat Model of Combined Diabetes and Alzheimer’s Disease. Frontiers in Neuroscience 2020, Volume 13 - 2019, doi:10.3389/fnins.2019.01400. PMC6985467 32038127

[216] Raghavan A; Shah ZA Sirtuins in neurodegenerative diseases: a biological-chemical perspective. Neurodegener Dis 2012, 9, 1–10, doi:10.1159/000329724. 22041967 PMC3254029

[217] Jin T; Zhang Y; Botchway BOA; Huang M; Lu Q; Liu X. Quercetin activates the Sestrin2/AMPK/SIRT1 axis to improve amyotrophic lateral sclerosis. Biomed Pharmacother 2023, 161, 114515, doi:10.1016/j.biopha.2023.114515. 36913894

[218] Dong X-W; Fang W-L; Li Y-H; Chai Y-R Epigallocatechin-Gallate: Unraveling Its Protective Mechanisms and Therapeutic Potential. Cell Biochemistry and Function 2025, 43, e70056, doi:10.1002/cbf.70056. 39915982

[219] Deng H; Mi MT Resveratrol Attenuates Aβ25–35 Caused Neurotoxicity by Inducing Autophagy Through the TyrRS-PARP1-SIRT1 Signaling Pathway. Neurochem Res 2016, 41, 2367–2379, doi:10.1007/s11064-016-1950-9. 27180189

[220] Leslie SN; Datta D; Wang M; van Dyck CH; Arnsten AFT; Nairn AC Biochemical characterization of age-related calcium-cAMP-PKA signaling dysregulation and its effect on tau pathology in rhesus monkey cortex. Alzheimer's & Dementia 2020, 16, e042017, doi:10.1002/alz.042017.

[221] Ahn JH; McAvoy T; Rakhilin SV; Nishi A; Greengard P; Nairn AC Protein kinase A activates protein phosphatase 2A by phosphorylation of the B56delta subunit. Proc Natl Acad Sci U S A 2007, 104, 2979–2984, doi:10.1073/pnas.0611532104. 17301223 PMC1815292

[222] Tsukamoto S; Huang Y; Umeda D; Yamada S; Yamashita S; Kumazoe M; Kim Y; Murata M; Yamada K; Tachibana H. 67-kDa laminin receptor-dependent protein phosphatase 2A (PP2A) activation elicits melanoma-specific antitumor activity overcoming drug resistance. J Biol Chem 2014, 289, 32671–32681, doi:10.1074/jbc.M114.604983. 25294877 PMC4239619

[223] Planel E; Yasutake K; Fujita SC; Ishiguro K. Inhibition of Protein Phosphatase 2A Overrides Tau Protein Kinase I/Glycogen Synthase Kinase 3β and Cyclin-dependent Kinase 5 Inhibition and Results in Tau Hyperphosphorylation in the Hippocampus of Starved Mouse *. Journal of Biological Chemistry 2001, 276, 34298–34306, doi:10.1074/jbc.M102780200. 11441005

[224] Sontag J-M; Sontag E. Protein phosphatase 2A dysfunction in Alzheimer’s disease. Frontiers in Molecular Neuroscience 2014, Volume 7 - 2014, doi:10.3389/fnmol.2014.00016. PMC3949405 24653673

[225] Parvathenani LK; Calandra V; Roberts SB; Posmantur R. cAMP delays beta-amyloid (25–35) induced cell death in rat cortical neurons. Neuroreport 2000, 11, 2293–2297, doi:10.1097/00001756-200007140-00045. 10923688

[226] Vilas-Boas F; Bagulho A; Tenente R; Teixeira VH; Martins G; da Costa G; Jerónimo A; Cordeiro C; Machuqueiro M; Real C. Hydrogen peroxide regulates cell adhesion through the redox sensor RPSA. Free Radic Biol Med 2016, 90, 145–157, doi:10.1016/j.freeradbiomed.2015.11.019. 26603095

[227] Gibbs CA; Fedoretz-Maxwell BP; MacNeil GA; Walsby CJ; Warren JJ Proximal Methionine Amino Acid Residue Affects the Properties of Redox-Active Tryptophan in an Artificial Model Protein. ACS Omega 2023, 8, 19798–19806, doi:10.1021/acsomega.3c01589. 37305310 PMC10249128

[228] Moss BL; Taubner L; Sample YK; Kazmin DA; Copié V; Starkey JR Tumor shedding of laminin binding protein modulates angiostatin production in vitro and interferes with plasmin-derived inhibition of angiogenesis in aortic ring cultures. International Journal of Cancer 2006, 118, 2421–2432, doi:10.1002/ijc.21674. 16380995

[229] Gundimeda U; McNeill TH; Elhiani AA; Schiffman JE; Hinton DR; Gopalakrishna R. Green tea polyphenols precondition against cell death induced by oxygen-glucose deprivation via stimulation of laminin receptor, generation of reactive oxygen species, and activation of protein kinase Cepsilon. Journal of Biological Chemistry 2012, 287, 34694–34708. 22879598 10.1074/jbc.M112.356899 PMC3464573

[230] Buryanovskyy L; Fu Y; Boyd M; Ma Y; Hsieh TC; Wu JM; Zhang Z. Crystal structure of quinone reductase 2 in complex with resveratrol. Biochemistry 2004, 43, 11417–11426, doi:10.1021/bi049162o. 15350128 PMC3650734

[231] Gould NL; Scherer GR; Carvalho S; Shurrush K; Kayyal H; Edry E; Elkobi A; David O; Foqara M; Thakar D, Specific quinone reductase 2 inhibitors reduce metabolic burden and reverse Alzheimer's disease phenotype in mice. J Clin Invest 2023, 133, doi:10.1172/jci162120. PMC10541198 37561584

[232] Hashimoto T; Nakai M. Increased hippocampal quinone reductase 2 in Alzheimer's disease. Neurosci Lett 2011, 502, 10–12, doi:10.1016/j.neulet.2011.07.008. 21803122

[233] Islam F; Shilton B. Insights into the cellular function and mechanism of action of quinone reductase 2 (NQO2). Biochem J 2025, 482, 309–324, doi:10.1042/bcj20240103. PMC12225741 40071447

[234] Hansen DV; Hanson JE; Sheng M. Microglia in Alzheimer's disease. J Cell Biol 2018, 217, 459–472, doi:10.1083/jcb.201709069. 29196460 PMC5800817

[235] Kim YA; Lim SY; Rhee SH; Park KY; Kim CH; Choi BT; Lee SJ; Park YM; Choi YH Resveratrol inhibits inducible nitric oxide synthase and cyclooxygenase-2 expression in beta-amyloid-treated C6 glioma cells. Int J Mol Med 2006, 17, 1069–1075. 16685418

[236] Ma T; Tan MS; Yu JT; Tan L. Resveratrol as a therapeutic agent for Alzheimer's disease. Biomed Res Int 2014, 2014, 350516, doi:10.1155/2014/350516. PMC4261550 25525597

[237] Regan P; Hole KL; Sero J; Williams RJ Epigallocatechin Gallate Modulates Microglia Phenotype to Suppress Pro-inflammatory Signalling Cues and Inhibit Phagocytosis. Mol Neurobiol 2024, 61, 4441–4453, doi:10.1007/s12035-023-03845-3. 38095777 PMC11236853

[238] Chen J; Zhou Y; Mueller-Steiner S; Chen LF; Kwon H; Yi S; Mucke L; Gan L. SIRT1 protects against microglia-dependent amyloid-beta toxicity through inhibiting NF-kappaB signaling. J Biol Chem 2005, 280, 40364–40374, doi:10.1074/jbc.M509329200. 16183991

[239] Sun GY; Chen Z; Jasmer KJ; Chuang DY; Gu Z; Hannink M; Simonyi A. Quercetin Attenuates Inflammatory Responses in BV-2 Microglial Cells: Role of MAPKs on the Nrf2 Pathway and Induction of Heme Oxygenase-1. PLoS One 2015, 10, e0141509, doi:10.1371/journal.pone.0141509. PMC4624710 26505893

[240] Wu L; Liu Y; He Q; Ao G; Xu N; He W; Liu X; Huang L; Yu Q; Kanamaru H, PEDF-34 attenuates neurological deficit and suppresses astrocyte-dependent neuroinflammation by modulating astrocyte polarization via 67LR/JNK/STAT1 signaling pathway after subarachnoid hemorrhage in rats. J Neuroinflammation 2024, 21, 178, doi:10.1186/s12974-024-03171-y. 39034417 PMC11264993

[241] Zhang W; Xiao D; Mao Q; Xia H. Role of neuroinflammation in neurodegeneration development. Signal Transduction and Targeted Therapy 2023, 8, 267, doi:10.1038/s41392-023-01486-5. 37433768 PMC10336149

[242] Zhang Q; Yang G; Luo Y; Jiang L; Chi H; Tian G. Neuroinflammation in Alzheimer's disease: insights from peripheral immune cells. Immun Ageing 2024, 21, 38, doi:10.1186/s12979-024-00445-0. 38877498 PMC11177389

[243] Dos Santos MG; Arbo BD; Hort MA Effects of quercetin and its derivatives in in vivo models of neuroinflammation: A systematic review and meta-analysis. Neural Regen Res 2026, 21, 1783–1792, doi:10.4103/nrr.Nrr-d-24-01175. 40145967 PMC12694622

[244] Serban MC; Sahebkar A; Zanchetti A; Mikhailidis DP; Howard G; Antal D; Andrica F; Ahmed A; Aronow WS; Muntner P, Effects of Quercetin on Blood Pressure: A Systematic Review and Meta-Analysis of Randomized Controlled Trials. J Am Heart Assoc 2016, 5, doi:10.1161/jaha.115.002713. PMC5015358 27405810

[245] Chen Y; He Y; Han J; Wei W; Chen F. Blood-brain barrier dysfunction and Alzheimer's disease: associations, pathogenic mechanisms, and therapeutic potential. Front Aging Neurosci 2023, 15, 1258640, doi:10.3389/fnagi.2023.1258640. PMC10679748 38020775

[246] Nehra G; Bauer B; Hartz AMS Blood-brain barrier leakage in Alzheimer’s disease: From discovery to clinical relevance. Pharmacology & Therapeutics 2022, 234, 108119, doi:10.1016/j.pharmthera.2022.108119. 35108575 PMC9107516

[247] Cortes-Canteli M; Mattei L; Richards AT; Norris EH; Strickland S. Fibrin deposited in the Alzheimer's disease brain promotes neuronal degeneration. Neurobiol Aging 2015, 36, 608–617, doi:10.1016/j.neurobiolaging.2014.10.030. 25475538 PMC4315732

[248] Yang AJT; Bagit A; MacPherson REK Resveratrol, Metabolic Dysregulation, and Alzheimer's Disease: Considerations for Neurogenerative Disease. Int J Mol Sci 2021, 22, doi:10.3390/ijms22094628. 33924876 PMC8125227

[249] Xia N; Förstermann U; Li H. Resveratrol and endothelial nitric oxide. Molecules 2014, 19, 16102–16121, doi:10.3390/molecules191016102. 25302702 PMC6270738

[250] Li Y; Zhou S; Li J; Sun Y; Hasimu H; Liu R; Zhang T. Quercetin protects human brain microvascular endothelial cells from fibrillar β-amyloid1–40-induced toxicity. Acta Pharmaceutica Sinica B 2015, 5, 47–54, doi:10.1016/j.apsb.2014.12.003. 26579424 PMC4629123

[251] Yang J; Chen W; Chen J; Xie D; Wang Y; Zhou J. Tea Polyphenol Epigallocatechin Gallate and the Gut–Health Axis: Unraveling Structural Characteristics, Metabolic Pathways, and Systemic Benefits. Advances in Nutrition 2025, 16, 100545, doi:10.1016/j.advnut.2025.100545. 41106481 PMC12651636

[252] Andersen JK; Davies KJ; Forman HJ Reactive oxygen and nitrogen species in neurodegeneration; Free Radic Biol Med. 2013 Sep;62:1–3. doi: 10.1016/j.freeradbiomed.2013.06.001. 23876468

[253] Crowell JA; Korytko PJ; Morrissey RL; Booth TD; Levine BS Resveratrol-associated renal toxicity. Toxicol Sci 2004, 82, 614–619, doi:10.1093/toxsci/kfh263. 15329443

[254] Shaito A; Posadino AM; Younes N; Hasan H; Halabi S; Alhababi D; Al-Mohannadi A; Abdel-Rahman WM; Eid AH; Nasrallah GK, Potential Adverse Effects of Resveratrol: A Literature Review. Int J Mol Sci 2020, 21, doi:10.3390/ijms21062084. 32197410 PMC7139620

[255] Andres S; Pevny S; Ziegenhagen R; Bakhiya N; Schäfer B; Hirsch-Ernst KI; Lampen A. Safety Aspects of the Use of Quercetin as a Dietary Supplement. Mol Nutr Food Res 2018, 62, doi:10.1002/mnfr.201700447. 29127724

